# Progress of infrared guided-wave nanophotonic sensors and devices

**DOI:** 10.1186/s40580-020-00222-x

**Published:** 2020-04-02

**Authors:** Yiming Ma, Bowei Dong, Chengkuo Lee

**Affiliations:** 1 Department of Electrical and Computer Engineering, National University of Singapore, Singapore, 117576 Singapore; 2 Center for Intelligent Sensors and MEMS (CISM), National University of Singapore, Singapore, 117608 Singapore; 3 NUS Suzhou Research Institute (NUSRI), Suzhou Industrial Park, Suzhou, 215123 China; 4 NUS Graduate School for Integrative Science and Engineering (NGS), National University of Singapore, Singapore, 117456 Singapore

**Keywords:** Nanophotonics, Biochemical/physical sensors, Guided-wave, Infrared

## Abstract

Nanophotonics, manipulating light–matter interactions at the nanoscale, is an appealing technology for diversified biochemical and physical sensing applications. Guided-wave nanophotonics paves the way to miniaturize the sensors and realize on-chip integration of various photonic components, so as to realize chip-scale sensing systems for the future realization of the Internet of Things which requires the deployment of numerous sensor nodes. Starting from the popular CMOS-compatible silicon nanophotonics in the infrared, many infrared guided-wave nanophotonic sensors have been developed, showing the advantages of high sensitivity, low limit of detection, low crosstalk, strong detection multiplexing capability, immunity to electromagnetic interference, small footprint and low cost. In this review, we provide an overview of the recent progress of research on infrared guided-wave nanophotonic sensors. The sensor configurations, sensing mechanisms, sensing performances, performance improvement strategies, and system integrations are described. Future development directions are also proposed to overcome current technological obstacles toward industrialization.

## Introduction

Internet of Things (IoT) has been attracting more and more interests because of its promising potential in the efficient use of resources for various applications ranging from environmental monitoring to industrial process control, and from homeland security to personal healthcare [1]. IoT has set high requirements on both the sensor networks and the communication systems. The sensor networks need to consist of numerous sensor nodes that detect a variety of external stimuli including biochemical molecules, temperature change, radiation, pressure, acceleration, rotation, etc. Correspondingly, the communication systems should be equipped with a high data transmission capacity to deal with the large volume of data collected by sensors [2]. While most of IoT systems are working in the electrical domain, i.e., the sensed parameters are converted into electrical sensory information and transmitted electrically [3–6], the optical (in particular nanophotonic) systems, where the sensory information and/or the transmitted signal are in the optical domain, become a complementary technology [7, 8].

Nanophotonics is the study of light and its interactions with matters at the nanoscale. Over the past decades, nanophotonics, especially silicon (Si) nanophotonics, has attracted great research interests because of its promising potential to meet the increasing demands for high data transmission capacity in communication systems [9]. One key driving force of Si nanophotonics is the fabrication compatibility with the mature complementary metal–oxide–semiconductor (CMOS) technology, which enables the manufacturing of photonic integrated circuits (PICs) at low costs and high volumes [10]. The 1.31 and 1.55 μm wavelengths are the two optimized wavelengths for single-mode fiber, at which zero group velocity dispersion and lowest optical loss are achieved, respectively. As a result, Si nanophotonic devices working in near-infrared (NIR) wavelength range at around 1.31 and 1.55 μm for communications were first developed [11–13]. Up to date, the commercialization of Si nanophotonics has been witnessed [14]. In addition, the wide transparency window of Si from 1.1 to 8 μm allows the exploration of nanophotonics at other wavelengths including the mid-infrared (MIR, 2–20 μm).

**Fig. 1:**
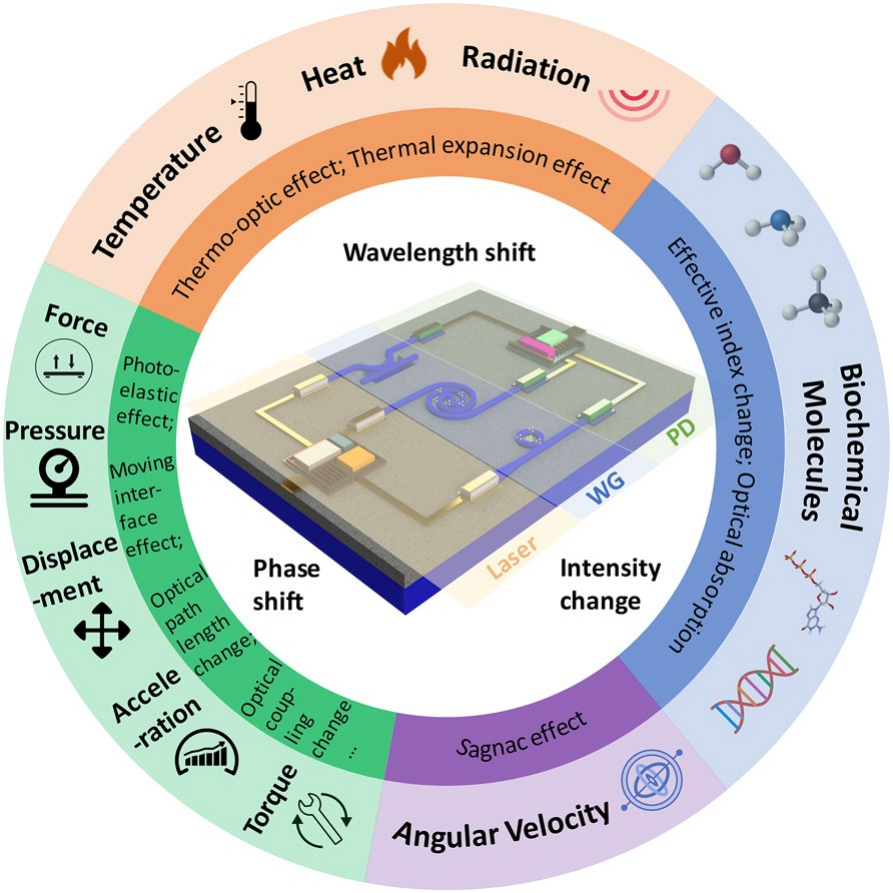
Schematic illustration of the sensing mechanisms of a typical on-chip integrated infrared guided-wave nanophotonic sensing system. *WG* guided-wave nanophotonic device, *PD* photodetector

During the development of nanophotonics for optical communication, its use for sensing applications is getting increasing attention, because the transmitted optical signal is sensitive to various external stimuli. Compared with other types of sensors, nanophotonic sensors provide advantages of high sensitivity, low limit of detection (LoD), low crosstalk, strong detection multiplexing capability, high stability, immunity to electromagnetic interference, small size and low cost [15, 16]. In comparison with the free-space configuration, the guided-wave configuration, where light is confined and guided by a waveguide, is more favorable in sensor miniaturization and their on-chip integration with other components including microfluidics, light sources, photodetectors, and optoelectronic circuits. In a representative on-chip guided-wave nanophotonic sensing system schematically illustrated in Fig. 1, the light beams ejected from a light source such as a laser are guided into nanophotonic devices and interact with various environmental stimuli, leading to changes in the optical signals due to the corresponding light–matter interaction effects. The changes in various optical properties, such as wavelength, phase, and intensity, are recorded by a photodetector and converted to electrical signals for analysis.

In this paper, we review the progress of infrared guided-wave nanophotonic sensors in the recent 20 years. Firstly, as these sensors are essentially used for biochemical sensing applications, the corresponding fundamental sensing mechanisms are introduced. Secondly, since the biochemical sensors start with and are mostly working in the NIR, an overview of the main configurations of NIR guided-wave nanophotonic biochemical sensors is presented with an emphasis on structures and corresponding sensing mechanisms. Several performance improvement approaches are also introduced. Thirdly, following the trend of nanophotonic biochemical sensors moving from the NIR to the MIR, we introduce the state-of-the-art MIR guided-wave nanophotonic biochemical sensors. We summarize the building blocks and functional devices developed in the MIR, which lay the foundation for the development of integrated sensors. The material platforms, sensor structures, and system integration schemes of the reported MIR sensors are introduced. The methodologies for multiplexed sensing in the MIR are highlighted. Fourthly, nanoplasmonics, as an emerging technology to enhance the performances of guided-wave nanophotonic biochemical sensors, is introduced. On top of biochemical sensing, the utilization of guided-wave nanophotonics for physical sensing, including the sensing of temperature, force, displacement, acceleration, angular velocity, torque, etc., is overviewed. In the end, we conclude the development of infrared guided-wave nanophotonic sensors to date and provide our points of view on their future development directions.

## Fundamentals of infrared guided-wave nanophotonic biochemical sensing

The detection principle of most infrared guided-wave nanophotonic biochemical sensors is based on evanescent field detection. While propagating through a dielectric waveguide, the light is confined by the total internal reflection happening at the interface between the waveguide core and the cladding medium with different refractive indices (RIs). As the light is reflected from the interface back into the waveguide, part of the electromagnetic field (a.k.a., evanescent field) penetrates into the cladding with an exponentially decaying field intensity with respect to the distance from the waveguide surface. The biochemical analytes perturb the evanescent field and modify the cladding RI and/or induce an additional optical absorption. The change in the cladding RI will primarily cause the shift of resonant peaks in photonic resonators, while the additional optical absorption reduces the light transmission intensity in waveguides. Accordingly, infrared guided-wave nanophotonic biochemical sensors can be generally classified into RI-based sensors and absorption-based sensors. As the native molecular properties are utilized, these sensors do not require external labeling molecules attached to the target molecule, i.e., they perform label-free detection.

**Fig. 2:**
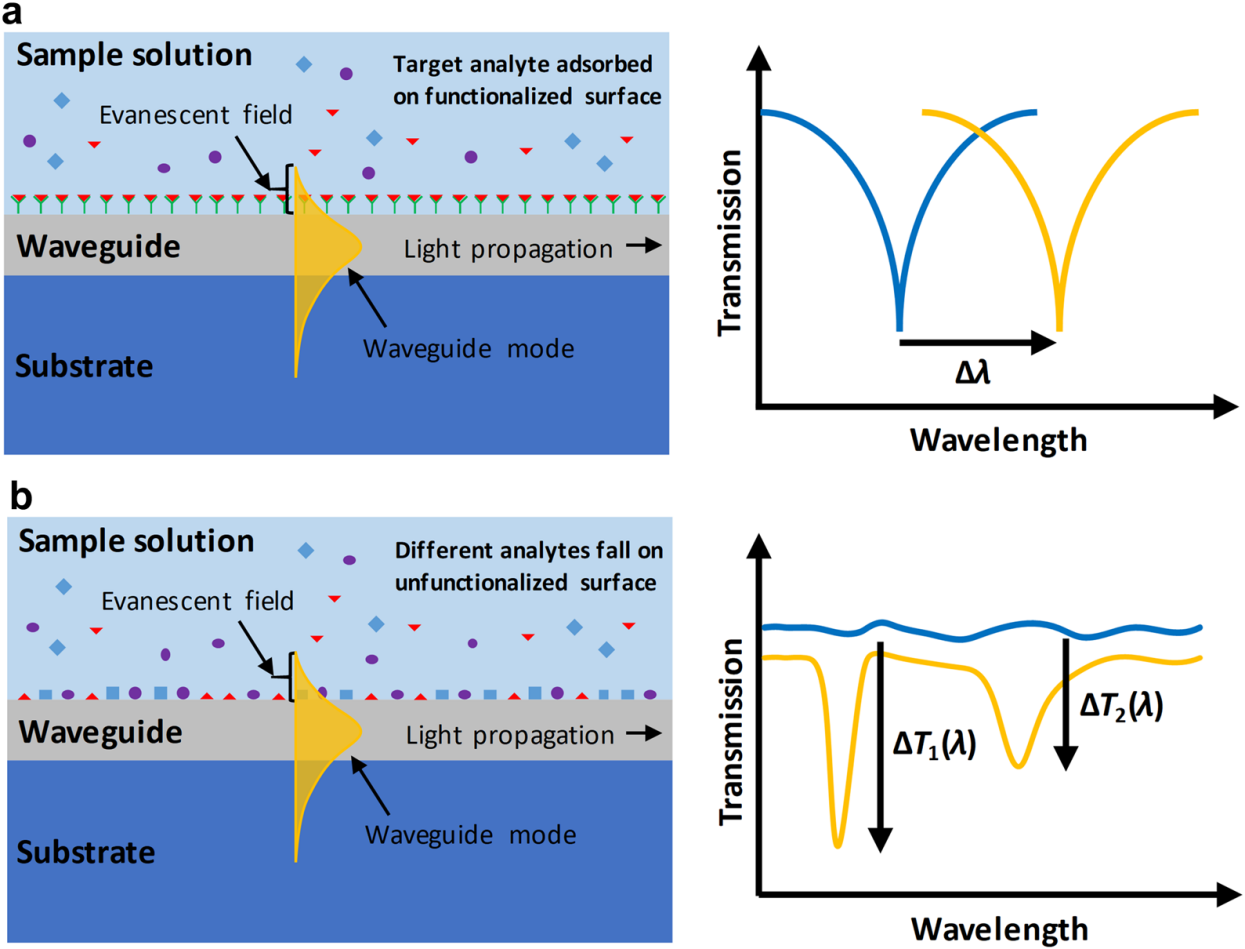
Schematic illustration of the sensing mechanisms of infrared guided-wave nanophotonic biochemical sensing based on evanescent field detection. **a** RI-based sensor with its surface functionalized to selectively adsorb analyte. The optical signal change in the sensor, e.g., the resonant wavelength shift in a resonator, is known to be induced by the absorbed analyte. **b** Absorption-based sensor without surface functionalization. The different analytes adsorbed on the sensor surface are distinguished by their induced characteristic absorption peaks at different wavelengths on the sensor transmission spectrum

Selectivity is one of the important criteria for the evaluation of sensor performance. The lack of selectivity is one major drawback of RI-based biochemical sensors, as different biochemical species register nearly indistinguishable shifts of RI when interacting with the evanescent field. Therefore, an additional process of sensor surface functionalization with specific receptors is required in order to realize selective adsorption. Hence, the target analytes can be identified, as illustrated in Fig. 2a. Conversely, absorption-based sensors leverage the characteristic (fingerprint) absorptions caused by the functional groups of the biochemical analytes. The characteristic absorption wavelengths are determined by the vibration frequencies of the functional groups. Consequently, different functional groups of unlike biochemical molecules possess different characteristic absorption bands, providing intrinsic molecular selectivity to absorption spectroscopy. Therefore, absorption-based sensors are free of surface functionalization, as shown in Fig. 2b.

In addition to selectivity, sensitivity and LoD are the other two critical figures of merit. According to the different ways the target molecules interact with the sensors, two definitions for sensitivity are used in biochemical sensing applications. One is the bulk sensitivity (*S*_bulk_), referring to the response of the sensor to a uniform change in the cladding, where the sensor surface is mostly not functionalized. The other one is the surface sensitivity (*S*_surf_), which assesses the sensor response to light–matter interactions at the adsorbed molecule layer on the sensor surface, where the adsorption is normally assisted by a receptor layer through the surface functionalization. For RI-based sensors, the bulk sensitivity is defined as the slope of wavelength (or phase) shift versus bulk refractive index unit (RIU) change:

1 $$S_{bulk} = \frac{{\Delta \lambda \left( {\Delta \phi } \right)}}{{\Delta n_{sample} }}$$

The surface sensitivity is defined slightly differently by replacing the RI of the sample (*n*_sample_) with the thickness of the adlayer on the sensor surface (*t*_adlayer_):

2 $$S_{surf} = \frac{{\Delta \lambda \left( {\Delta \phi } \right)}}{{\Delta t_{adlayer} }}$$

For absorption-based sensors, the sensitivity refers to bulk sensitivity in most cases as no surface functionalization is implemented, and is defined as the change in absorbance (or transmittance) divided by the change in sample concentration:

3 $$S_{bulk} = \frac{{\Delta R \left( {\Delta T} \right)}}{{\Delta c_{sample} }}$$

The LoD is classified into system LoD (sLoD) and intrinsic LoD (iLoD). sLoD is defined as:

4 $$sLoD = \frac{3\sigma }{S}$$

where *σ* is the system noise floor, and *S* is the corresponding bulk or surface sensitivity depending on whether the LoD is referring to bulk or surface sensing. *σ* and sLoD depend significantly on the performance of the measurement system including light sources, photodetectors and readout circuits. iLoD is introduced as a substitute for resonance-based sensors, which is only dependent on the intrinsic characteristics of the sensors, i.e., the resonance linewidth, and defined as:

5 $$iLoD = \frac{\lambda }{{{\text{Q}} \times {\text{S}}}}$$

where *λ*, *Q*, and *S* are the resonant wavelength, quality factor (Q factor), and bulk or surface sensitivity of the sensor, respectively. It is worth noting that the comparison of sensitivities and LoDs among different reported works can be a challenge because of the non-uniform units. For the bulk sensitivity and the bulk LoDs of RI-based sensor, although a normalized unit of RIU can be employed, it is not normalized to other factors such as the light–matter interaction length and the working wavelength. For the other cases, various parameters are used to characterize the sample concentration, such as mass per volume, mass per surface area, molar mass, etc., leading to different units of the derived sensitivities and LoDs.

## NIR guided-wave nanophotonic biochemical sensors

Following the development of optical communication, the research of utilizing guided-wave nanophotonic devices for biochemical sensing applications also start from the NIR. In addition to Si [17], sensors fabricated with a variety of CMOS-compatible materials, including Si_3_N_4_ [18], SiO_2_ [19], and Hydex [20], have been demonstrated. Some materials that are not compatible with the CMOS process are also employed to construct sensors, such as chalcogenide (ChG) glasses [21–23], polymers [24–26], and even liquid [27]. This section mainly focuses on the sensors based on CMOS-compatible materials, as they enable low-cost mass production and ease the integration with CMOS electrical circuits.

### Sensor configurations

The representative guided-wave nanophotonic structures, that have been reported and widely used as label-free biochemical sensors at the operating wavelengths of NIR, are reviewed in the following sub-sections.

#### Interferometer

**Fig. 3:**
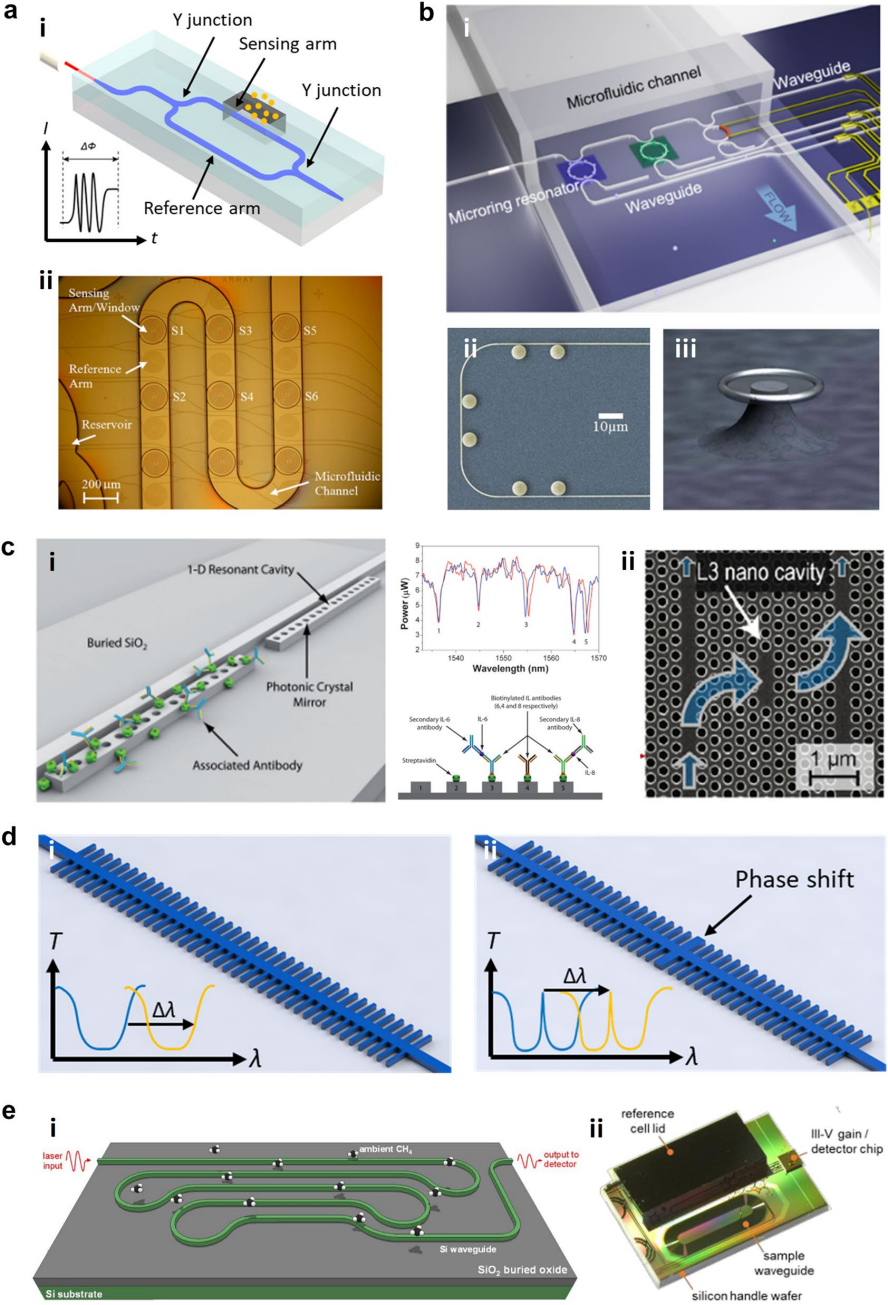
Basic configurations of NIR guided-wave nanophotonic biochemical sensors. **a** Interferometer: (i) schematic illustration of MZI biochemical sensor; (ii) microscopic image of MZI biochemical sensor array integrated with microfluidic channel (Reproduced with permission from [30]). **b** Microcavity resonator: (i) schematic illustration of MRR biochemical sensor integrated with microfluidic channel (Reproduced with permission from [31]); (ii) false-color SEM image of cascaded microdisk resonators on single bus waveguide (Reproduced with permission from [40]); (iii) SEM image of microtoroid resonator (Reproduced with permission from [19]). **c** Photonic crystal (PhC): (i) PhC nanobeam cavity biochemical sensor array (Reproduced with permission from [45]); (ii) SEM image of L3 PhC nanocavity accessed by W1 PhC waveguide (Reproduced with permission from [52]). **d** Bragg grating. Schematic illustrations of (i) Bragg grating; (ii) phase-shited Bragg grating. **e** Channel waveguide: (i) schematic illustration of methane absorption spectroscopy on a Si ridge waveguide (Reproduced with permission from [62]); (ii) integrated Si nanophotonic on-chip sensor (Reproduced with permission from [63])

Among various types of interferometers, Mach-Zehnder interferometer (MZI) is the most common one used for interferometric sensing in the infrared. In a typical MZI sensor schematically illustrated in Fig. 3a (i), the guided light is split by a Y-junction into two paths, one of which passes through the sample and is regarded as the sensing arm, and the other is used as the reference arm. The evanescent field of the sensing arm interacts with the sample and senses the RI change at the surface, resulting in an optical phase shift. After a certain distance, the two beams are recombined by another Y-junction and cause constructive or destructive interference at the output, where the intensity *I* depends on the phase difference Δ*ϕ*. And the phase difference Δ*ϕ* is determined by the effective index difference Δ*n*_eff_ between sample and reference arms:

6 $$I \propto \cos \left( {\Delta \phi \left( t \right)} \right)$$

7 $$\Delta \phi \left( t \right) = \frac{2\pi L}{\lambda }\Delta n_{eff}$$

where *L* is the light–matter interaction length in the sensing arm.

Densmore et al. [17] developed the first silicon-on-insulator (SOI) photonic wire waveguide based MZI sensor in 2006. The sensor was used for sucrose sensing, showing a sensitivity of 300 × 2π rad/RIU with an interaction length of 1.5 mm. Later, they reported another MZI sensor utilizing double-spiral waveguide geometry. The interaction length was increased to 2 mm while the sensor compactness was maintained. As a result, a higher sucrose sensitivity of 460 × 2π rad/RIU was realized [28]. The sensor response to molecular adsorption was also examined by using the biotin-streptavidin binding reaction. A minimum detectable surface coverage of ~ 6 pg/mm^2^ was achieved [29]. Afterward, they realized multiplexed real-time and label-free molecular detection by employing an array of double-spiral MZI sensors (Fig. 3a (ii)). A surface sLoD of ~ 0.25 pg/mm^2^ immunoglobulin G (IgG) surface coverage was achieved [30].

#### Microcavity resonator

Optical microcavity resonators are also widely utilized for RI-based biochemical sensing applications because they can enhance optical path lengths while maintaining small footprints. In a microcavity resonator, light propagating in the bus waveguide or tapered fiber is coupled into the microcavity via the evanescent field. The coupled light propagates in the microcavity in the form of whispering gallery modes (WGMs) or circulating waveguide modes, generating resonances at specific frequencies. The light keeps resonating inside the microcavity until adsorption and dissipation phenomena end up diminishing the energy resonating. The resonant wavelength *λ* is given by:

8 $$\lambda = 2\pi r\frac{{n_{eff} }}{m}$$

where *r* is the radius of the resonator, *n*_eff_ is the effective index of the resonator, and *m* is the resonance order. The analytes change *n*_eff_ in the evanescent field, and consequently shift the resonant peaks, which can be monitored by scanning the wavelength or by measuring the intensity at a single wavelength.

As the light propagates in the microcavity with multiple rounds, the light–matter interaction length is not simply determined by the physical dimensions of the guided-wave structure, but by the characteristic time of the energy stored inside the resonator, which is characterized by the Q factor. The Q factor is a dimensionless parameter determined by the ratio of the stored energy in the cavity to the energy dissipated, and is calculated by dividing the resonant wavelength to its full width at half maximum (FWHM):

9 $$Q = \frac{\lambda }{{\Delta \lambda_{FWHM} }}$$

The effective length *L*_eff_ of the resonator is directly linked to the Q factor as follows:

10 $$L_{eff} = Q\frac{\lambda }{2\pi n}$$

where *n* is the refractive index of the resonator.

Various types of planar microcavity resonators have been implemented for biochemical sensing applications, such as microring, microdisk, and microtoroid resonators, as shown in Fig. 3b (i–iii), respectively. Among them, the MRR is the most widely employed configuration. Numerous MRR biochemical sensors based on Si [31], Si_3_N_4_ [32], and Hydex [20], have been reported. In some of the reported devices, straight sections were introduced between the two half rings, forming a racetrack shape, so as to have better control of the coupling between the microcavity and the bus waveguide [33, 34]. Utilizing micro-ring/racetrack resonator arrays, multiplexed sensing of biomolecules was also demonstrated [35–37]. Zang et al. [38] developed an MRR biochemical sensor integrated with a low dark current germanium (Ge) photodetector, which enhanced the signal-to-noise ratio (SNR) and improved the system stability. Homogeneous sensing with a sensitivity of ~ 18.8 nm/RIU and a sLoD of 3.50 × 10^−5^ was demonstrated. Laplatine et al. [39] developed a system-level architecture for Si nanophotonic biochemical sensors. Si dies of photodetector-integrated MRR arrays were packaged through Fan-Out Wafer-Level-Packaging, which could reduce the Si die size while increasing the effective surface for microfluidic integration and electrical interconnects. Compared with MRR, the WGM in microdisk resonator travels near the outside perimeter and only contacts one sidewall, leading to a lower scattering loss and a higher Q factor. Additionally, smaller radius can be realized in microdisk resonator, which not only leads to lower surface LoD but also results in a wider free spectral range (FSR) that favors the integration of multiple microdisk resonators on a single bus waveguide for multiplexed sensing. As shown in Fig. 3b (ii), Schmidt et al. [40] cascaded six disks on a single bus waveguide. The radius of each disk are slightly different to avoid overlapping FSRs and ensure their resonant peaks occupy different locations within the spectra. The multiplexed biosensing capability was assessed by performing a modified sandwich assay involving well-characterized biomolecules.

In addition to RI-based sensors, microcavity resonators have also been employed to enhance the performances of absorption-based sensors. The absorption induced by the analyte alters the extinction ratio and the Q factor of the transmission resonance. Nitkowski et al. [41] demonstrated on-chip laser absorption spectroscopy using silicon MRRs with a radius of 100 μm and a Q factor of ~ 120,000, which corresponds to an effective free space path length of ~ 5 mm. *N*-methylaniline with an absorption peak near 1500 nm was chosen as the analyte. Less than 2 nL of fluid was needed to cover a single ring resonator. As a result, its absorption spectrum was measurable. Hu et al. [22] developed another *N*-methylaniline absorption sensor based on a ChG glass microdisk resonator. A sLoD of 0.02 cm^−1^ was achieved, which represents threefold improvement as compared to their previous result attained with a straight waveguide sensor, while the physical device length is reduced by 40-fold [21]. Armani et al. [19] demonstrated a D_2_O detector employing a microtoroid resonator working at a wavelength of around 1300 nm. The Q factor of the resonator increases with the increasing concentration of D_2_O in H_2_O due to the weakened optical absorption. By monitoring the Q factor, a concentration of 1 part per million by volume (ppmv) has been detected.

#### Photonic crystal

Photonic crystal (PhC) is another common configuration used to construct a resonant cavity. A PhC is composed of periodic dielectric nanostructures, forming a periodic variation in the refractive index. The periodicity blocks a range of wavelengths, giving rise to a photonic bandgap in the transmission or reflection spectrum of the PhC [42]. By introducing a defect into the PhC, a defect mode is formed and resonantly confined in the defect, which leads to a sharp peak within the bandgap. The strong optical confinement in the defect enhances the light–matter interaction. Consequently, a small volume of analyte surrounding the defect is able to induce a noticeable shift of the resonant wavelength [43].

Planar PhCs can be classified into one-dimensional (1D) and two-dimensional (2D) according to their periodicities along one or two axes, respectively. The 1D PhC as the simplest PhC architecture is also named as PhC nanobeam. Mandal et al. [44] reported a 1D PhC resonator array, where five 1D PhC resonators with slightly different central defect cavity spacings are evanescently coupled to a single bus waveguide. The bulk RI sensing of CaCl_2_ was demonstrated with a sensitivity of 130 nm/RIU. Later on, the 1D PhC resonator array was employed to demonstrate multiplexed biomolecular sensing. As shown in Fig. 3c (i), three of the five resonators were functionalized with antibodies to interleukin 4, 6, and 8, while the rest two are functionalized with glutaraldehyde and streptavidin served as controls for non-specific analyte adsorption. The initial spectrum and the spectrum after introducing interleukin 6 and 8, followed by sequential association of secondary antibodies corresponding to each of these interleukins, were measured. A significant resonance shift was only observed in the two resonators functionalized with anti-interleukin 6 and 8, while the other resonances do not shift appreciably [45]. Chen et al. [46] demonstrated a PhC nanobeam cavity with an as-fabricated Q factor of 50,000. After the chemical functionalization, a surface sLoD of 1.5 parts per billion (ppb) was achieved for methyl salicylate.

The typical structures of 2D PhC cavities can be classified into four types: H*m* type modifying *m* lattice points; L*n* type removing *n* lattice points in one line; ring type removing some lattice points to form one or more ring; hetero type spanning a large number of lattice points with different lattice constants, holes sizes, holes locations, or shapes [47]. Chen et al. [48] reported the first PhC cavity-based biochemical sensor in 2004, which is an H0 cavity fabricated on the SOI. The bulk RI sensing was demonstrated using a silicone fluid, showing a sensitivity of ~ 160 nm/RIU and a sLoD of lower than 0.002 RIU. Later on, various H0 cavity-based biochemical sensors have been demonstrated [49–51]. Dorfner et al. [52] experimentally evaluated the biosensing performances of different types of 2D PhC cavities. The cavities were accessed from W1 line defect PhC waveguides (Fig. 3c (ii)). When introducing a line defect by removing lattice points along one direction, a guided band arises within the photonic bandgap. As a result, light can propagate through the line defect with good confinement provided by the PhC on both sides. The width modulated cavity showed the highest surface iLoD due to its high Q factor. The H1 cavity showed the highest sensitivity but the lowest iLoD, while the L3 cavity has both moderate sensitivity and iLoD. Hsiao et al. [53] studied biosensors employing PhC-based hexagonal nanoring resonator and dual-nanoring resonators [54]. Ho et al. [55] proposed a biosensor utilizing PhC-based hexagonal triple-nanoring resonators. Sünner et al. [56] demonstrated a gas sensor based on hetero cavity. A bulk sensitivity of 80 nm/RIU was realized.

#### Bragg grating

Bragg grating, a fundamental component for wavelength selection in optical communication, has also been utilized for biochemical sensing applications. Similar to 1D PhC, a Bragg grating is a structure with a periodic modulation of the effective index in the light propagation direction by alternating the material with different indices or physical dimensions of the waveguide, as schematically illustrated in Fig. 3d (i). The guided light is reflected at each index-changed boundary. The repeated boundaries multiply the distributed reflection, resulting in a stop band in the transmission spectrum. The center wavelength of the stop band, namely the Bragg wavelength *λ*_B_, is given as:

11 $$\lambda_{B} = 2\varLambda n_{eff}$$

where Λ is the grating period, *n*_eff_ is the effective index of Bragg grating. By monitoring the shift of the stop band edge, biochemical sensors based on Bragg gratings have been demonstrated [57–59]. If a phase shift region is introduced in the middle of the gratings, as illustrated in Fig. 3d (ii), it constructs a cavity with the two Bragg reflector mirrors on both sides, inducing a resonant peak within the stop band, which has also been utilized for RI change monitoring [40, 60].

#### Channel waveguide

Although microcavity resonators are able to enhance the light–matter interaction length while maintaining the small footprint of absorption-based sensors, the basic channel waveguide is still the most popular structure for absorption-based sensing. The monitoring of extinction ratio or Q factor in microcavity resonators requires wavelength sweeping and additional calculation, while the absorption sensing using channel waveguide can be simply performed by monitoring the transmission intensity at a single wavelength. In addition, the resolution of a microcavity resonator is determined by its FSR, while the resolution of a channel waveguide is only limited by the linewidth of the laser.

Ryckeboer et al. [61] demonstrated NIR absorption spectroscopy of glucose, whose peak absorption wavelength is 1590 nm, based on a 10-mm-long SOI spiral rib waveguide. A sLoD of 1 mM was achieved. Green’s group from IBM leveraged SOI ridge waveguide for methane absorption spectroscopy around 1651 nm. As shown in Fig. 3e (i), the waveguide was designed in a spatially efficient paperclip configuration. The 10-cm-long waveguide occupies merely 16 mm^2^. Allan-variance analysis indicated a bandwidth-normalized sLoD of 772 ppmv Hz^−1∕2^. Therefore, it is calculated that sLoD below 100 ppmv could be achieved for integration times > 1 min, reaching ∼ 20 ppmv at 103 s [62]. Very recently, the same group integrated a Si nanophotonic sensor on-chip with a III–V/Si external cavity laser and a photodetector, as shown in Fig. 3e (ii). Using a waveguide sensing element with 30 cm length, a sLoD of 92.8 ppmv Hz^−1∕2^ was realized, which is comparable to commercially available tunable diode laser absorption spectroscopy (TDLAS) sensors [63]. Katiyi et al. [64] proposed an SOI strip rib waveguide structure for label-free on-chip overtone spectroscopy in NIR. High order modes were excited and guided in the waveguide, enhancing the light–matter interaction. Through the spectral scanning over the absorption dip of first N–H overtone in the NIR, *N*-methylaniline and aniline were precisely distinguished without any surface treatment of the waveguide. Du et al. [23] demonstrated an on-chip spectroscopic sensor where a ChG glass waveguide served as both the broadband supercontinuum (SC) light source and the evanescent sensing element. A pump source centered at 1560 nm was coupled into the GeSbSe waveguide, where the SC was generated. A maximum SC spectral span from 1380 to 2050 nm was realized, which covers the C–H overtone absorption peak centering at 1695 nm. As a result, the absorption sensing of CHCl_3_ was successfully conducted.

### Performance improvement strategies

As mentioned above, the integration of sensors with lasers and photodetectors can improve the performance of the integrated sensing system. On top of it, the performance improvement is also able to be realized through the optimization of the sensor itself, including new waveguide geometries, and different optical mode polarizations or working wavelengths. Various reported performance improvement strategies are reviewed in this section.

#### 1310 nm working wavelength

Most of the NIR sensors work around 1550 nm, where the water absorption is strong and predominantly limits the signal intensity, the Q factors, and consequently the LoD of aqueous sensing. This problem can be well addressed by changing the working wavelength to another common wavelength, i.e., 1310 nm. It was observed that water absorption is approximately 10 times lower around 1310 nm compared to that around 1550 nm [65]. Assuming an ideal Fabry–Perot cavity and light traveling entirely in the water, with no other loss mechanism other than water absorption, the iLoD is calculated to be 2.40 × 10^−4^ RIU at 1550 nm and 3.14 × 10^−5^ RIU at 1310 nm [66].

Schmidt et al. [40] demonstrated several SOI biochemical sensors working at 1310 nm, including MRRs in transverse electric (TE) and transverse magnetic (TM) modes, and a phase-shifted Bragg grating in TM mode. Compared with the same structures working at 1550 nm, higher Q factors and lower iLoDs were observed. Melnik et al. [67] demonstrated a polyimide-based MZI biosensor at a central wavelength of 1310 nm. Human IgG detection was performed, showing a surface sLoDs of 3.1 nM and 0.03 nM by label-free and labeled methods, respectively.

#### TM mode

**Fig. 4:**
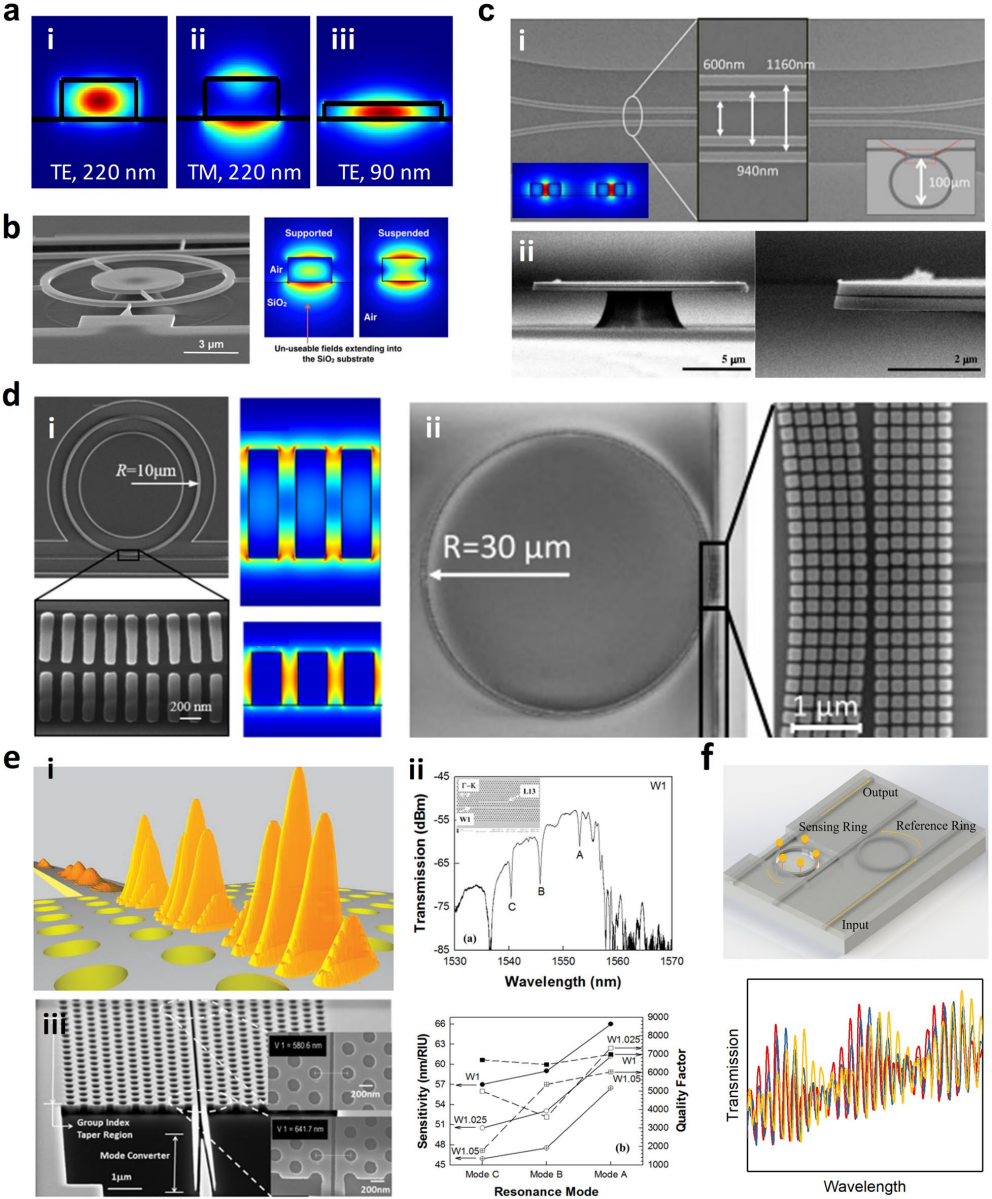
Performance improvement strategies for guided-wave nanophotonic biochemical sensors. **a** TM mode and thinner waveguide. Mechanisms indicated by the simulated electric field intensity distributions of (i) TE mode in 220 nm × 500 nm waveguide; (ii) TM mode in 220 nm × 500 nm waveguide; (iii) TE mode in 90 nm × 800 nm waveguide. **b** Suspended waveguide. Example using suspended MRR (Reproduced with permission from [74]). **c** Slot waveguide: (i) lateral slot (Reproduced with permission from [79]); (ii) horizontal slot (Reproduced with permission from [87]). **d** (i) Subwavelength grating waveguide (Reproduced with permission from [90]); (ii) Subwavelength multibox waveguide (Reproduced with permission from [92]). **e** Slow light effect: (i) schematic illustration of slow light enhancement mechanism (Reproduced with permission from [94]); (ii) slow light enhanced sensitivity in L*n* PhC microcavity side coupled to W1 PhC waveguide (Reproduced with permission from [103]); (iii) absorption enhancement in PhC slot waveguide (Reproduced with permission from [110]). **e** Vernier light effect. Schematic illustration of sensor configuration employing Vernier effect and typical Vernier spectra in RI sensing

Figure 4a (i) and (ii) shows the simulated electric field intensity distributions of the TE and TM modes propagating in a 220 nm × 500 nm waveguide. As can be clearly seen, a larger part of the field intensity is in the cladding and substrate in the TM mode, offering stronger light–matter interaction. The TM mode has been adopted in numerous RI-based biochemical sensors in configurations of MZI [17, 28–30], MRR [29, 68], microdisk resonator [69, 70], and phase-shifted Bragg grating [40]. Numbers of absorption-based sensors have also adopted the TM mode, with configurations of channel waveguide [21, 62, 63] and MRR [41].

#### Thinner waveguide

In addition to the TM mode, the use of thinner waveguide also helps to increase the ratio of field intensity in the cladding, as the optical confinement is weakened (Fig. 4a (iii)). Fard et al. [71] investigated an ultra-thin TE MRR sensor using the thinnest thickness of 90 nm offered by the standard multi-project wafer (MPW) foundries. A sensitivity of 133 nm/RIU and an iLoD of ~ 5 × 10^−4^ RIU was achieved in the bulk sensing of glucose. Later, they investigated the optimized waveguide thickness to achieve the maximum bulk sensitivity using TM mode, which was 165 nm. The closest thickness offered by MPW foundry services of 150 nm was chosen. The bulk sensitivity was measured to be 270 nm/RIU, whereas a similar resonator sensor with a 220 nm thick waveguide demonstrated a bulk sensitivity of approximately 200 nm/RIU [72].

#### Suspended waveguide

Although the utilization of TM mode and thinner waveguide can increase the ratio of the field intensity in the cladding and substrate, the part penetrating into the substrate does not interact with the analyte. Therefore, another straightforward method to increase the overlap between the optical field and the analyte is introducing suspended waveguides. Wang et al. [73] demonstrated a suspended ultra-small microdisk resonator with a radius of 0.8 µm sitting on a SiO_2_ pedestal, showing a bulk sensitivity of 130 nm/RIU and a bulk iLoD of 8 × 10^−4^ RIU. Afterward, Gaur et al. [74] demonstrated a suspended TM MRR sensor (Fig. 4b), showing a threefold enhancement in the bulk sensitivity and a twofold increase in the surface sensitivity, compared with a supported MRR. The suspension strategy has also been employed in PhC-based sensors, where the substrate undercut can be conveniently performed utilizing the air holes of PhC as the releasing holes [52, 56].

#### Slot waveguide

A slot waveguide consists of two high index rails separated by a low index slot, which is normally the cladding. The optical field is strongly confined within the slot. As a result, the light–matter interaction can be significantly strengthened compared with conventional strip waveguides, leading to an improved sensing performance [75].

Barrios et al. [32] pioneered the development of slot waveguide based biochemical sensors by using a Si_3_N_4_-based MRR. The sensor operated at 1.3 µm and achieved a bulk sensitivity of 212 nm/RIU and a bulk sLoD of 2 × 10^−4^ RIU. Afterward, numerous MRR sensors with the slot waveguide design have been reported [76–78]. Zhang et al. [79] investigated a new approach for RI sensing using SOI slot waveguide racetrack resonators. The detection is implemented by analyzing the giant shift of the transmission spectrum envelope. The slot waveguide cross-section, as well as the racetrack coupler, were designed to lead to a V-shaped transmission spectrum (Fig. 4c (i)). By monitoring the full spectrum envelope peak position, a high bulk sensitivity of 1300 nm/RIU was achieved.

The slot waveguide design has also been employed in MZI sensors. Tu et al. [80] developed a thermally independent Si_3_N_4_ slot waveguide MZI sensor, with a bulk sensitivity of 1730 × 2π rad/RIU and a bulk sLoD of 1.29 × 10^−5^ RIU. Later, they further investigated the biosensing capability using the biotin–streptavidin binding, showing a surface sensitivity of ∼ 4.55π/(ng mm^−2^) and a surface sLoD down to 18.9 fM or 1 pg/ml of streptavidin solution. The specific detection and quantification of the methylation of death-associated protein kinase (DAPK) gene were also investigated, showing the capability of quantifying and discriminating at a concentration as low as 1 fmol/µl or 1 nM [81]. Sun et al. [82] developed an MZI liquid sensor employing an ultra-compact double-slot hybrid plasmonic (DSHP) waveguide as the active sensing arm. By introducing a DSHP waveguide with two open nanoslots among a Si ridge waveguide and two silver strips, an extremely high optical confinement was achieved, showing a sensitivity as high as 1061 nm/RIU.

Slot PhC cavities combine the advantages of the spatial confinement by slot and the temporal confinement by PhC cavity. Hetero [83, 84] and L*n* [85] slot PhC cavities have been demonstrated for biochemical sensing applications. The slot design has also been adopted in a phase-shifted Bragg grating sensor [86].

Apart from the common lateral slot, Lee et al. [87] demonstrated label-free biosensing using a horizontal air-slot SiN_x_ microdisk resonator as shown in Fig. 4c (ii), obtaining a Q factor of 7000 in TM mode with a surface sensitivity of 2.5 nm/(µg/ml) and a surface sLoD of 30 ng/ml for the biotin–streptavidin interaction.

#### Subwavelength grating waveguide

Subwavelength grating (SWG) waveguide is an emerging and appealing strategy to tailor the guided-wave optical properties. Although SWG waveguide is also a periodic structure similar to Bragg grating, the period Λ is much smaller than the Bragg condition, i.e., $\varLambda \ll \lambda /\left( {2n_{eff} } \right)$. As a result, light can propagate in SWG waveguides with a low loss comparable to propagating in conventional strip waveguides. Compared with the mode in strip waveguide, a larger ratio of the mode in SWG waveguide penetrates into the evanescent field, due to the lower effective index. Moreover, most of the optical field is concentrated in the low-index region. As a result, a stronger light–matter interaction can be achieved in SWG waveguides, leading to a better sensing performance.

Flueckiger et al. [88] experimentally demonstrated the first SWG-based MRR biosensor in 2016. A Q factor of 7000, a bulk sensitivity of 490 nm/RIU, a bulk sLoD of 2 × 10^−6^ RIU, and a bulk iLoD of 4.5 × 10^−4^ RIU, were achieved. The primary bottleneck for the Q factor of SWG MRR is the large bend loss. It was reported that the use of trapezoidal pillars instead of conventional rectangular pillars as the building block of SWG MRRs can significantly reduce the bend loss and therefore increase the Q factor of SWG MRRs, by creating an asymmetric effective index profile [89]. Utilizing the trapezoidal pillar geometry as shown in Fig. 4d (i), Yan et al. [90] demonstrated an SWG-based MRR biosensor with an improved Q factor of 9100 and as a result a lower a bulk iLoD of 3.9 × 10^−4^ RIU. To further improve the iLoD, Huang et al. [91] optimized the Q factor of an SWG racetrack resonator in the TM mode to 9800 and realized an improved bulk iLoD of 3.71 × 10^−4^ RIU. Very recently, Luan et al. [92] proposed a subwavelength multibox waveguide MRR, which combines SWG and slot structures, as shown in Fig. 4d (ii). The multibox structure significantly enhances the light–matter interaction, resulting in a high bulk sensitivity of 579.5 nm/RIU. In addition to MRRs, the SWG design has also been adopted in the MZI sensors [93].

#### Slow light effect

In addition to increasing the ratio of optical field in the cladding by the above-mentioned methods, slow light is another promising technology that can be leveraged to enhance the light–matter interaction. As schematically illustrated in Fig. 4e (i), slow light with remarkably low group velocity spatially compresses optical energy, as a result, enhances the light–matter interaction [94, 95]. Slow light on-chip can be realized using various periodic structures. The periodicity folds back the guided-mode band at the Brillouin zone edge and opens up a bandgap, which splits the band into two bands and flattens the bands near their band edges. Consequently, strong slow light effect occurs near the band edge [96]. The above-mentioned line defect PhC waveguide used to couple light into the PhC microcavity is the most popular periodic structure to realize the slow light effect [97–99].

Chen’s group from the University of Texas at Austin investigated the slow light engineering in the sensors based on the configuration of L*n* PhC microcavity side coupled to a W1 line defect PhC waveguide. Closer to the transmission band edge of the W1 PhC waveguide, the light propagates slower in the waveguide and interacts more sufficiently with the microcavity, which enhances the coupling efficiency between the microcavity and the waveguide [100–102]. As a result, the resonance mode closest to the W1 PhC waveguide transmission band edge provides the strongest light-matter interaction and thus the highest sensitivity, as illustrated in Fig. 4e (ii) [103]. The multiplexed specific label-free detection of lung cancer cell lysates was demonstrated using an array of L13 PhC microcavity with its resonant wavelength close to the band edge of the coupled W1 PhC waveguide. A surface sLoD down to 2 cells per microliter was achieved [104]. To further improve the sensing performance, they introduced defect holes into the PhC microcavity, which increases the ratio of electric field energy existing outside of the dielectric structure and enhances the light–matter interaction. A bulk sensitivity of 112 nm/RIU and a surface sLoD of 1 fM were achieved, both surpass the values obtained in their other sensors without the defect holes [105].

Besides coupling with PhC cavities, the band edge fringes of the W1 PhC waveguide has also been utilized for biochemical sensing, where the slow light effect is strong. By monitoring the wavelength shift of the band edge fringes, a bulk sensitivity of 174.8 nm/RIU and a bulk sLoD of 1.55 × 10^−4^ RIU were achieved. In the label-free anti-bovine serum albumin (anti-BSA) biosensing, a surface sensitivity of 0.706 nm/ng/mm^2^ and a surface mass density sLoD of 2.1 pg/mm^2^ were estimated [106].

In addition to the line defect PhC waveguide, PhC nanobeam is another periodic structure that offers slow light effect. Qin et al. [107] embedded a slow light nanobeam into an MZI to construct a label-free biosensor. Compared with conventional MZI biosensors, a fivefold higher bulk sensitivity (103 nm/RIU) was achieved with a 400 times smaller sensing arm length. Wang et al. [108] proposed a PhC slot nanobeam slow light waveguide, which combines the light–matter interaction enhancement effects of slow light and slot. A high bulk sensitivity of 900 nm/RIU was achieved. Applying PhC design to MRR not only raises the slow light effect but also increases the area for the light–matter interaction. Lo et al. [109] reported a PhC MRR and investigated its use for label-free biosensing. More than twofold improvement in bulk and surface sensitivities was achieved compared to conventional MRR sensors. A degree of slow light effect also exists near the band edge of the periodic Bragg gratings and is naturally leveraged when monitoring the band edge shift in sensing [57–59].

In addition to the above-mentioned RI-based sensors, slow light effect has also been utilized for absorption-based sensors by Chen’s group. As shown in Fig. 4e (iii), they demonstrated a 300-µm-long PhC slot waveguide, which combines the light–matter interaction enhancement effects of slow light and slot. Through tailoring the lattice constant of the PhC and the radius of the air hole, the band edge of the waveguide was designed to overlap with the absorption peak of xylene at 1697 nm [110] and of methane at 1665.5 nm [111], respectively. A xylene concentration down to 100 ppb in water and a methane concentration of 100 ppm in nitrogen were measured, respectively. They also demonstrated the multiplexed detection of xylene and trichloroethylene (TCE) in water by two 300-µm-long W1 PhC waveguides connected by a multimode interference (MMI) splitter and a Y-junction combiner. The band edges of the two waveguides are designed to overlap with the absorption peaks of xylene and TCE located at 1674 and 1644 nm, respectively. A sLoD of 1 ppb for xylene and a sLoD of 10 ppb for TCE in water were achieved [112].

#### Vernier effect

The Vernier effect is a method commonly used in calipers and barometers to enhance the accuracy of measurement instruments. It consists of two scales with different periods, of which one slides along the other one. The overlap between lines on the two scales is used to perform the measurement. Figure 4f illustrates how this concept can be applied to an MRR sensor. Two MRRs with different optical roundtrip lengths (and as a result different FSRs) are cascaded. As the transmission spectrum of the cascaded MRRs is the product of the transmission spectra of the individual MRRs, it only exhibits peaks at wavelengths where the resonance peaks of the respective MRRs have overlap, and the peak height is determined by the amount of overlap. Therefore, a major peak together with some minor peaks are presented at the output, showing a Vernier FSR equal to the least common multiple of the FSRs of the individual MRRs. The sensing ring with an open window acts as the sliding part of the Vernier-scale, while the reference ring with an upper cladding acts as the fixed part. When the RI above the sensing ring changes, the major peak shifts (∆*λ*_maj_) equals to multiple FSRs of the reference ring ($\Delta \lambda_{FSR}^{ref}$), i.e., $\Delta \lambda_{maj} = m\Delta \lambda_{FSR}^{ref}$. In this way, the Vernier sensor has an ultra-high sensitivity *S* which is given by:

12 $$\lambda = \frac{{\lambda_{maj} }}{{n_{eff} }}\frac{{\Delta \lambda_{FSR}^{ref} }}{{\Delta \lambda_{FSR}^{ref} - \Delta \lambda_{FSR}^{sen} }} = S_{0} M$$

where $\Delta \lambda_{FSR}^{sen}$ is the FSR of the sensing ring, *S*_0_ is the actual sensitivity of a single ring. Therefore, a sensitivity enhancement factor of $\frac{{\Delta \lambda_{FSR}^{ref} }}{{\Delta \lambda_{FSR}^{ref} - \Delta \lambda_{FSR}^{sen} }}$ is achieved [113].

Claes et al. [114] developed two cascaded MRRs with 2.5 mm physical roundtrip length and a reduced footprint by spiral design. A bulk sensitivity of 2169 nm/RIU was achieved, while that of a single ring was 76 nm/RIU. By introducing a curve fitting method to remove the limitation by the FSR of the reference ring, an improved bulk sLoD of 8.3 × 10^−6^ RIU was realized. Hu et al. [115] reported two cascaded MRRs, where the sensing ring was suspended by removing the SiO_2_ underneath. A bulk sensitivity of 4.6 × 10^5^ nm/RIU and a bulk iLoD of 4.8 × 10^−6^ RIU were achieved. He’s group from Zhejiang University demonstrated two cascaded racetrack resonators, showing a bulk sensitivity of 1300 nm/RIU compared to 62 nm/RIU for a single ring [116]. Later, they proposed cascaded double-ring sensors operating in TM mode. An ultrahigh bulk sensitivity of 24,300 nm/RIU was achieved, compared to the sensitivity of 165 nm/RIU using a single ring [117]. Moreover, they demonstrated three cascaded MRRs with a sensitivity of 5866 nm/RIU. The measurement range was significantly improved by 24.7 times compared with the traditional two cascaded MRRs, where the measurement range was limited by the FSR of the sensing ring [118].

In addition to cascaded MRRs, Jiang et al. [119] reported a Vernier effect sensor based on cascaded MZI and MRR. A bulk sensitivity of 21,500 nm/RIU was achieved, which is significantly higher than that of a single MZI sensor (2870 nm/RIU). A biosensing application was also demonstrated by monitoring the interaction between goat and antigoat IgG pairs. An amount of 1 ng/ml IgG resulted in 0.035 nm and 0.5 nm wavelength shift for the MZI sensor and the MZI-MRR sensor, respectively. The lowest detectable IgG concentration was 0.29 ng/ml and 0.1 ng/ml for the MZI sensor and the MZI-MRR sensor, respectively. The Vernier effect was also utilized in two cascaded MZIs, showing an 8.38 times enhancement in both the surface sensitivity and the surface sLoD [80].

## MIR guided-wave nanophotonic biochemical sensors

### Building blocks and material platforms

**Fig. 5:**
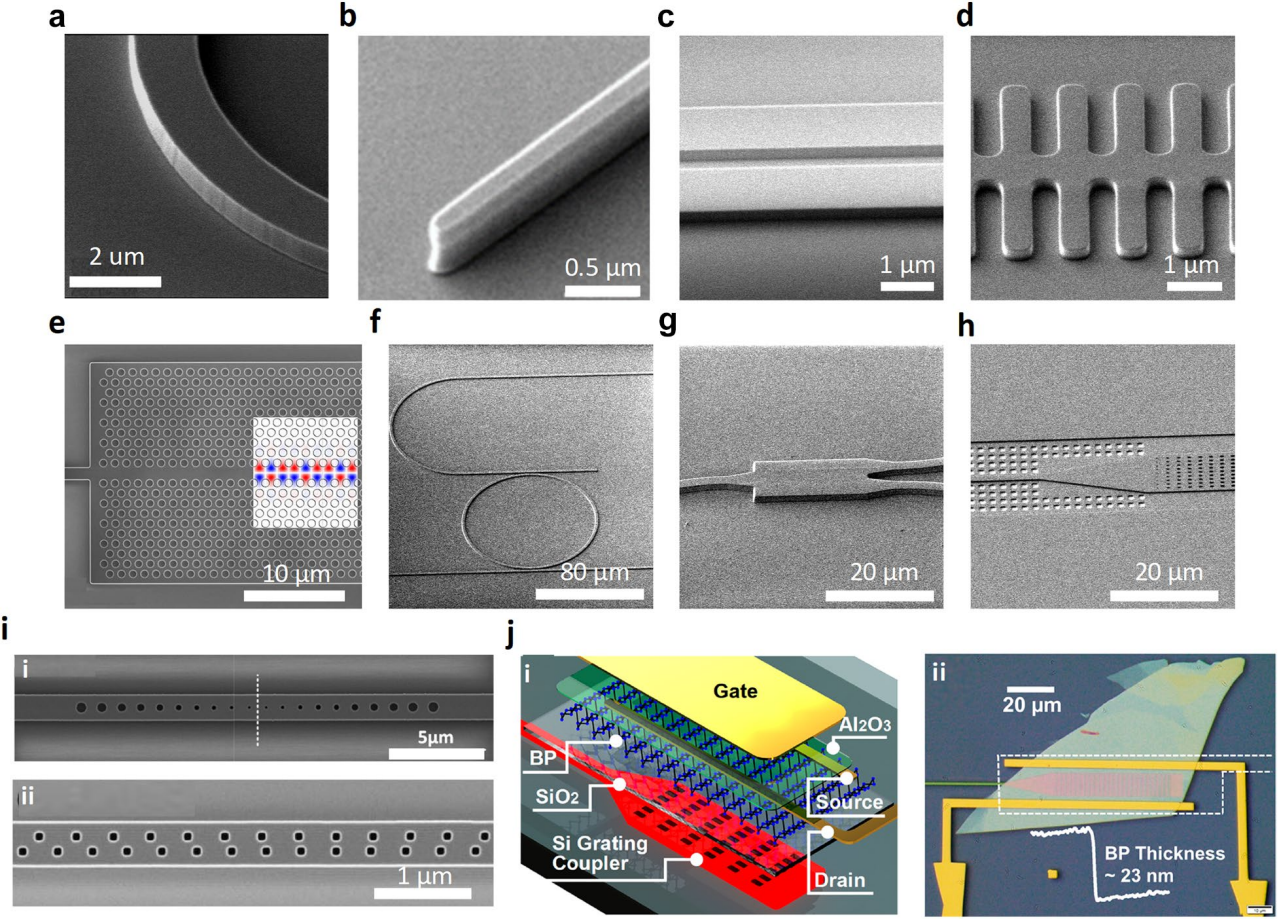
MIR nanophotonic building blocks.**a** Waveguide bends (Reproduced with permission from [105]). **b** Tapered fiber-to-chip coupler (Reproduced with permission from [105]). **c** Directional coupler. **d** 1D grating slow light waveguide (Reproduced with permission from [96]). **e** 2D PhC slow light waveguide (Reproduced with permission from [97]). **f** Add-drop filter (Reproduced with permission from [143]). **g** Multimode interferometer (Reproduced with permission from [143]). **h** Grating coupler with releasing holes. **i** Nanocavities, (i) with tapered hole radius (Reproduced with permission from [127]), (ii) aperiodic deterministic nanobeam (Reproduced with permission from [128]). **j** Black-phosphorus-based waveguide integrated on-chip photodetector, (i) schematic of the device, (ii) optical image. (Reproduced with permission from [131])

Despite many absorption-based guided-wave nanophotonic biochemical sensors demonstrated in the NIR, some drawbacks are inevitable. Firstly, the number of molecular fingerprints is limited in the NIR spectrum. Only sensing of substances that are rich in the C–H bond such as methane (CH_4_), xylene (C_8_H_10_), and *N*-methylaniline (C_7_H_9_N) has been demonstrated. Secondly, the absorption originated from these NIR fingerprints is weaker compared to the absorption caused by the MIR counterparts, leading to a theoretically inferior sensing performance. The MIR spectrum covering 2–20 µm in the electromagnetic (EM) wave spectrum is an ideal wavelength range for absorption-based guided-wave nanophotonic biochemical sensors [120]. It contains most of the molecular fingerprints of biochemical bonds, including C–H, C–O, C–C, C=C, O–H, N–O, etc. [121]. These biochemical bonds are prominent indicators for the analysis of trace gases, industrial gases, and human breath. These benefit environmental monitoring, industrial control, and healthcare applications. To realize miniaturized MIR guided-wave nanophotonic biochemical sensing systems, fundamental photonic building blocks have been developed during the past 10 years. Compared to the NIR, the critical dimensions in MIR waveguide design are larger so that it posts a less stringent requirement on fabrication. Furthermore, the waveguide loss caused by the sidewall roughness scattering is lower in the MIR which is a result suggested by the Rayleigh scattering. A MIR waveguide with low propagation loss of around 2 dB/cm in the wavelength range of 3.68–3.88 µm has been reported [121]. Compact bends with a 10 µm radius (Fig. 5a) and tapered fiber-to-chip couplers with 200 nm taper width (Fig. 5b) were demonstrated in the same work with a low bend loss of 0.02 dB/90° and a fiber-to-chip coupling efficiency of around -6 dB/facet. Directional couplers (DCs) (Fig. 5c), which are used for light routing and power splitting, have been investigated to achieve broadband operation which is desired in the MIR spectroscopy [122, 123]. Slow light structures including 1D grating waveguides (Fig. 5d) and 2D PhC waveguides (Fig. 5e) were developed for a stronger light–matter interaction [96, 97]. Tunability in slow-light devices was realized by thermo-optic (TO) effect. Add-drop filters for multiplexing and de-multiplexing (Fig. 5f), multimode interferometers for power splitting (Fig. 5g), and grating couplers for fiber-to-chip coupling (Fig. 5h) have also been reported in several works [124–126]. Optical nanocavity is a period structure with an artificial bandgap which provides a strong resonance with low damping. In the MIR, the coexistence of air and dielectric modes in single nanocavity was demonstrated (Fig. 5i (i)) [127]. It is expected the air mode will work with a high sensitivity in sensing because the electric field has a large overlap with the analyte. Meanwhile, the dielectric mode is rather inert against the analyte because the electric field is concentrated in the waveguide core. Consequently, the dielectric mode serves as a reference signal while the air mode acts as the probing signal. Figure 5i (ii) shows the other type of MIR nanocavity featuring a deterministic aperiodic PhC nanobeam [128]. Several adjustable multiple mode-matched resonances exist in the structure that will benefit integrated nonlinear optics. On top of the passive devices, active components are also necessary for the MIR sensing systems. Modulators working at 3.8 µm based on both TO effect [129] and free-carrier dispersion effect [130] have been reported with kHz and MHz modulation speed respectively. As shown in Fig. 5j, the on-chip integration of waveguide and photodetector up to 4 µm has been realized by using the low dimensional black phosphorus as the photodetection material [131]. A satisfactory responsivity of 2 A/W at 4.03 µm has been reported. More comprehensive review articles on the MIR photonic devices can be found in [132–134].

**Fig. 6:**
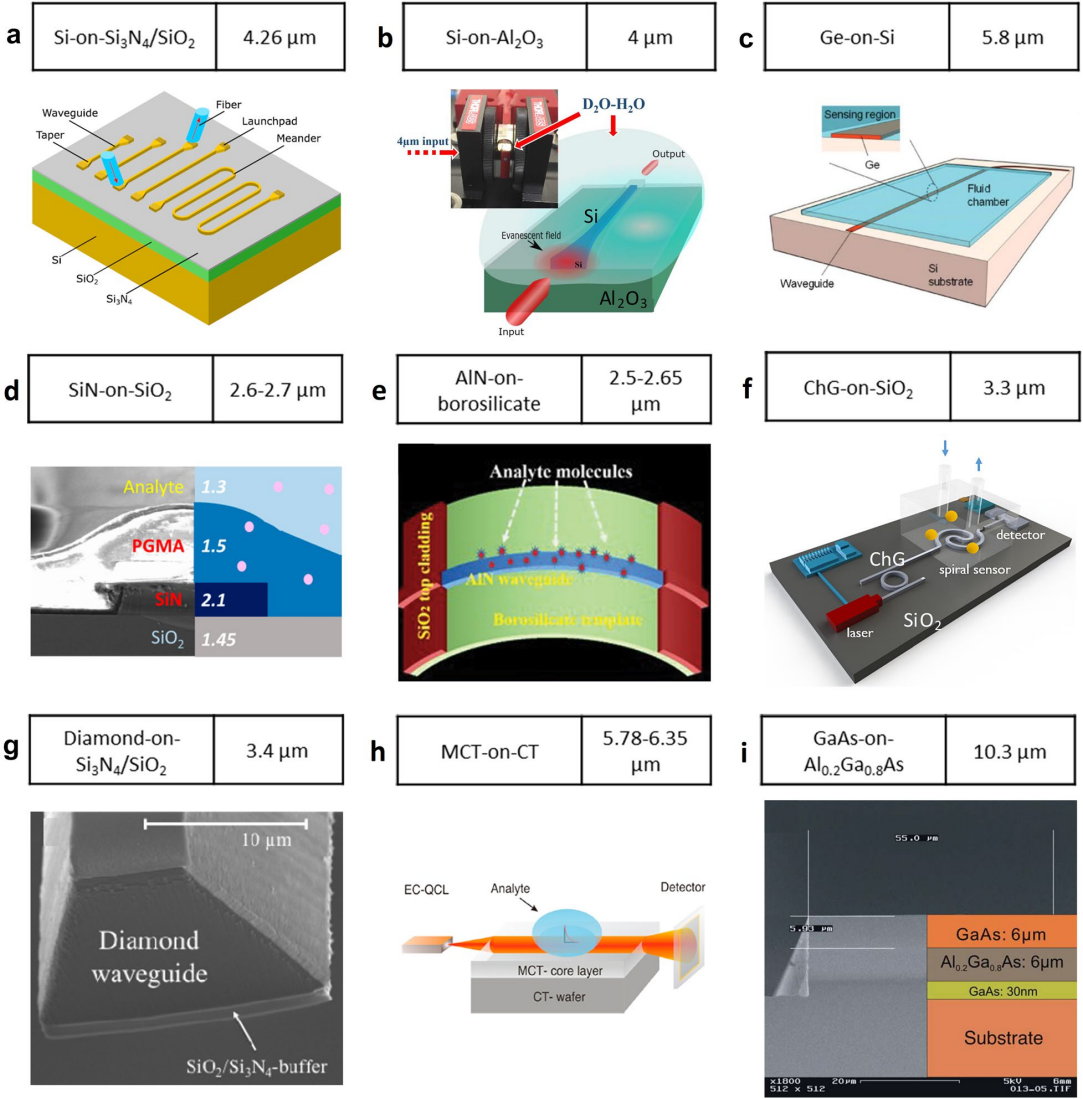
Typical material platforms for MIR guided-wave nanophotonic biochemical sensors. **a** Silicon-on-insulator (Reproduced with permission from [145]). **b** Silicon-on-sapphire (Reproduced with permission from [146]). **c** Germanium-on-silicon (Reproduced with permission from [147]). **d** Silicon nitride-on-insulator (Reproduced with permission from [148]). **e** Aluminum nitride-on-borosilicate (Reproduced with permission from [149]). **f** Chalcogenide-on-insulator (Reproduced with permission from [150]). **g** Diamond-on-insulator (Reproduced with permission from [151]). **h** Mercury cadmium telluride-on-cadmium telluride (Reproduced with permission from [152]). **i** Gallium arsenide-on-Al_0.2_Ga_0.8_As(Reproduced with permission from [153])

Several CMOS-compatible material platforms have been investigated for MIR applications, including the SOI [135, 136], silicon nitride-on-insulator (SNOI) [137], silicon-on-sapphire (SOS) [138], germanium-on-insulator (GOI) [139], germanium-on-silicon (GOS) [140], germanium-on-silicon nitride (GOSN) [141], silicon–germanium alloy (SiGe)-on-Si (SGOS) [142], and aluminum nitride-on-insulator (AlNOI) [143, 144]. Nonetheless, there are limited material choices among CMOS-compatible material so that the whole MIR spectrum cannot be fully utilized. Meanwhile, the integration of the sensor with on-chip light sources and photodetectors is also a challenge [134]. To extend the working wavelength and provide more convenient integration approaches, non-CMOS-compatible materials are also studied, including ChG, diamond, and III–V material. Figure 6 shows some typical material platforms for MIR guided-wave nanophotonic biochemical sensors. The SOI is mostly adopted due to its maturity (Fig. 6a). A CO_2_ sensor has been demonstrated by using a meander waveguide structure with a detection limit of 500 ppm [145]. However, the bottom cladding SiO_2_ starts absorbing beyond 4 µm. To solve the problem, the bottom SiO_2_ cladding has been replaced by Al_2_O_3_ which is transparent up to 5.5 µm (Fig. 6b). A D_2_O sensor was demonstrated based on this platform [146]. To further extend the platform transparency window, the GOS platform which is theoretically transparent up to 8 µm is adopted (Fig. 6c). Using this platform, the cocaine detection with a detection limit of 1.2 µg/ml and a fast response time of 1.2 min was demonstrated at the working wavelength of 5.8 µm [147]. Nitride materials also catch great research interests due to their excellent optical nonlinearity and CMOS-compatibility (Fig. 6d, e). Both the SiN and AlN are leveraged as MIR waveguide materials for biochemical sensing [148, 149] while they are also capable of working in the ultra-violet spectrum. It is worth noting that the AlN-on-borosilicate waveguide in [149] possesses flexibility enabled by thinning of the borosilicate substrate. Such technology shows the promise of nanophotonic waveguides for future wearable applications. Besides the CMOS-compatible materials, some non-CMOS-compatible materials offer irreplaceable advantages. The ChG can potentially cover the whole MIR spectrum and it can integrate with photodetectors at ease. Figure 6f shows an aerosol sensing system that was proposed in 2018. It features the on-chip integration of lasers, spiral sensors, and photodetectors. An add/drop filter was employed for wavelength sweeping [150]. The diamond possesses a wide transparency window in the MIR and a strong chemical and physical stability. A diamond waveguide has been successfully fabricated and applied for isopropyl alcohol (IPA, C_3_H_8_O) sensing at 3.4 µm with a detection limit of 200 pl (Fig. 6g) [151]. The mercury cadmium telluride-on-cadmium telluride (MCT-on-CT) (Fig. 6h) and GaAs-on-Al_0.2_Ga_0.8_As (Fig. 6i) platforms are also investigated since they have the potential for long-wave infrared (LWIR) applications. The MCT-on-CT platform has been demonstrated for acetone (C_3_H_6_O) sensing in the 5.78–6.35 µm wavelength range with a detection limit of 90 pl [152], envisaging the possibility of interfacing a waveguide with an MCT detector. The GaAs-on-Al_0.2_Ga_0.8_As platform works at 10.3 µm for acetic anhydride (C_4_H_6_O_3_) sensing with a detection limit of 0.05 pl, showing the potential of monolithically integrating waveguides and lasers using the common III-V material platform [153].

### Sensor configurations and system integration

**Fig. 7:**
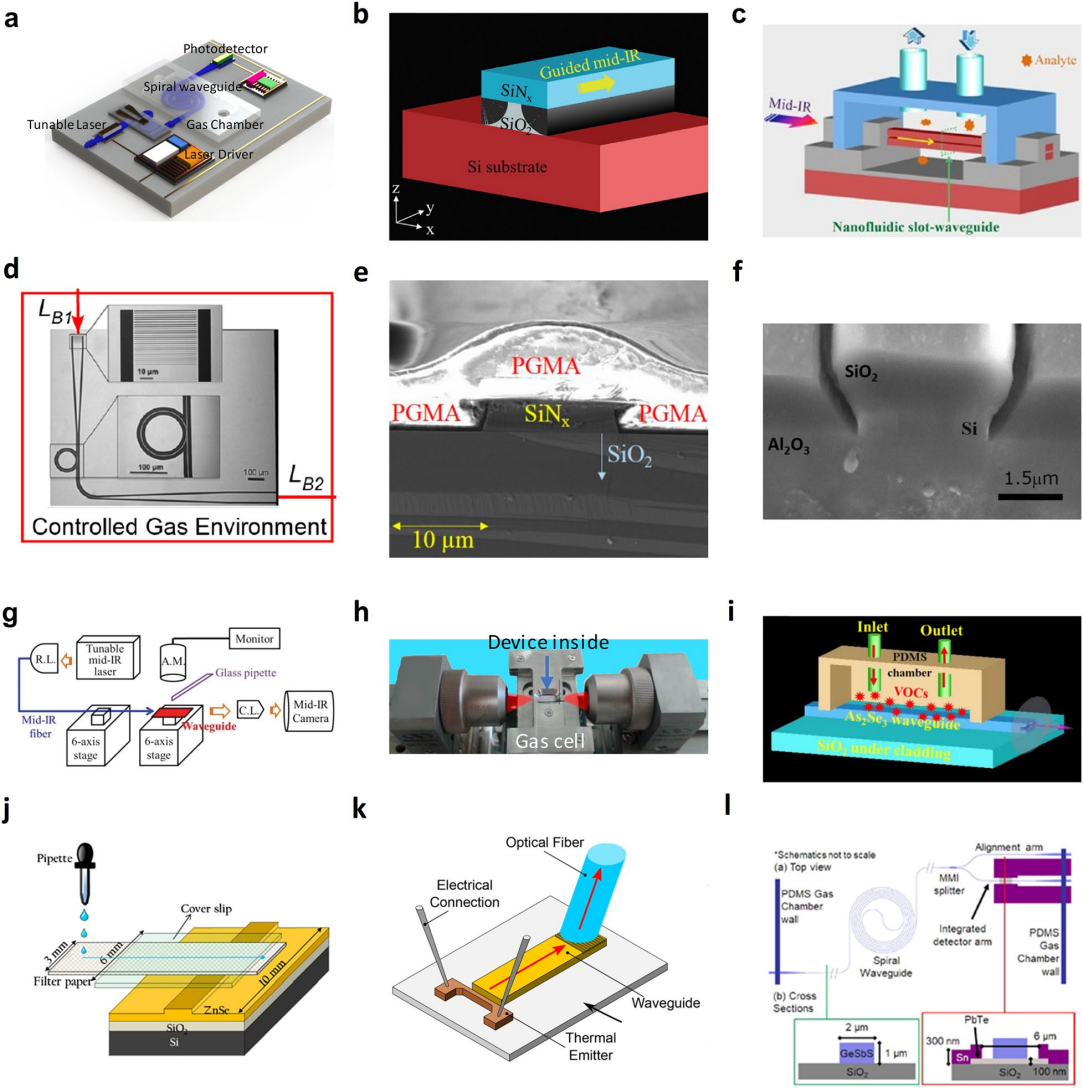
Device configurations and system integration of MIR guided-wave nanophotonic biochemical sensors. **a** Spiral waveguide (Reproduced with permission from [154]). **b** Pedestal waveguide (Reproduced with permission from [155]). **c** Slot waveguide (Reproduced with permission from [156]). **d** Micro-ring resonator (Reproduced with permission from [157]). **e** Enhancement layer coating (Reproduced with permission from [148]). **f** Side-trench waveguide (Reproduced with permission from [146]). **g** Manual liquid drop delivery using glass pipette (Reproduced with permission from [155]). **h** Tiny gas cell with MIR transparent windows (Reproduced with permission from [158]). **i** PDMS microfluidic chamber (Reproduced with permission from [159]). **j** Paper-based microfluidics (Reproduced with permission from [161]. **k** Integrated thermal emitter as light source (Reproduced with permission from [163]). **l** Chalcogenide waveguide sensor integrated with PbTe photodetector (Reproduced with permission from [164])

In nanophotonic biochemical sensors, it is usually desired to obtain a strong light–matter interaction. The most common approach is to fold the waveguide by using a spiral waveguide structure (Fig. 7a) [154]. With proper design, a 4-mm-long waveguide can be accommodated in an area of 0.068 mm^2^, providing a satisfactory interaction length while maintaining a small footprint. Pedestal waveguide structures where the rectangular waveguide is mechanically supported by a narrow pillar beneath are also used (Fig. 7b) [155]. The waveguide bottom surface is exposed to the analytes to enhance the light–matter interaction. A dry glucose detection limit of < 150 mg/dl was experimentally demonstrated, showing an improvement of 70% compared to a conventional ridge waveguide. In slot waveguides, light propagates in the narrow slot (< 100 nm) sandwiched by two dielectric waveguides. A High electric filed intensity is achieved because the light power is concentrated in the slot. In 2014, a horizontal air slot (< 100 nm) sandwiched by the top and bottom dielectric Si waveguides was demonstrated (Fig. 7c) [156]. The analytes fill in the slot where light is propagating to maximize the light–matter interaction through the increased spatial overlap. The sensitivity is increased by 50 times when compared to the evanescent-wave sensing. Using resonator structure is another effective method to enhance the light–matter interaction. On resonance, light is trapped in the resonator with a longer photon lifetime so that the effective propagation length is elongated. A ring resonator fabricated on the SOS platform was used for N_2_O sensing at 4.5 µm with a detection limit of 5000 ppmv (Fig. 7d) [157]. Unlike spiral waveguide sensors and slot waveguide sensors where data analysis is performed by studying the intensity change at the analytes’ fingerprint, the resonance spectrum is necessary for resonator-based guided-wave nanophotonic biochemical sensors so that the Q factor can be extracted. Since the Q factor is determined by the loss in the resonator system, the additional loss introduced by the analytes can be derived from the change in the Q factor [41]. The analyte concentration can then be derived from the measured additional loss. Besides tailoring the waveguide structures, an enhancement layer can be coated on the waveguide surface. The enhancement layer can selectively (e.g. polyethylenimine (PEI) absorbing CO_2_) or non-selectively (e.g. Porous Si, graphene) absorb analyte molecules to increase the analyte concentration locally around the waveguide surface which has strong interaction with the evanescent wave. A poly(glycidyl methacrylate) (PGMA) layer was coated on the SiN waveguide for water sensing (Fig. 7e) [148]. A 7.6 times enhancement of sensitivity was experimentally demonstrated as a result of the swelling up of PGMA after exposure to water.

Stronger light–matter interactions sometimes can be undesirable. For instance, sensing D_2_O in water at 4 µm requires a weak light–matter interaction. With an absorption coefficient of 144 cm^−1^ in water and 18,000 cm^−1^ in D_2_O, the propagating light can be completely absorbed by introducing even a small amount of mixture. To weaken the light–matter interaction, a side-trench waveguide was proposed in 2016. As shown in Fig. 7f, only the two small trenches beside the waveguide sidewalls are exposed for analytes while all the rest of the spaces are filled by the SiO_2_ [146]. Using this technique, the detection of 2% D_2_O in water was achieved.

Advances in the sensing system have also been witnessed. Regarding the methodology of delivering analytes, in the early years, the guided-wave nanophotonic biochemical sensors were placed on a multi-axis stage in a common waveguide testing system. The liquid analyte was manually dropped to the sensing region using a glass pipette (Fig. 7g) [155]. For gas sensing, the nanophotonic biochemical sensors were enclosed in a customized gas cell with a top window for viewing, and two side windows for coupling (Fig. 7h). The light was coupled into and out from the waveguide sensing using MIR objective lenses [158]. To more accurately control the analyze flow, a polydimethylsiloxane (PDMS) chamber was bonded to the nanophotonic biochemical sensor chip (Fig. 7i) [159]. A microfluidic system was embedded in the PDMS chamber so that the analyte can be precisely directed to the desired sensing area without any unwanted interaction with the chip that introduces perturbations. In order to achieve a strong PDMS chamber bonding, the surface of the photonic chip should be treated by plasma in advance to allow more bond formations during the bonding process. However, this technique has a major drawback when it is applied for gas sensing [160]. The PDMS physically absorbs most of the gases, leading to desorption when the gas supply is off. Another smart method for liquid analyte delivery was demonstrated (Fig. 7j) [161, 162]. A paper can be patterned to form paper-based fluidics and put on the nanophotonic biochemical sensor chip. By leveraging the capillary force, the liquid can be guided to the well-defined sensing region through the paper-based fluidics even when it is dropped with poor spatial accuracy.

The monolithic integration of the waveguide sensor with light sources and photodetectors is anticipated towards miniaturized biochemical sensing systems. However, limited progress has been made. Although III–V materials are promising to fulfill this goal through wafer-bonding, the requirement of thick high-quality III–V thin film for MIR applications is a challenge. In 2018, a thermal emitter as a MIR light source was successfully integrated with a Si waveguide [163]. Figure 7k shows that the MIR light generated by the blackbody radiation can be coupled to the waveguide. The output light is coupled out of the chip to an optical fiber through a grating coupler. Using this system, a 10% CO_2_ detection has been demonstrated. Recently in 2019, a ChG waveguide CH_4_ sensor integrated with a PbTe photodetector was demonstrated (Fig. 7l) [164]. The integrated CH_4_ sensor works at 3.3 µm and achieves a detection limit of 1% by volume. The maximum sensitivity of 330 ppmv was expected after optimization.

### Analysis approach for mixture sensing

Versatile data analysis approaches are available in the MIR absorption sensing. In the NIR RI sensing, the only variable in the sensing system is the refractive index change induced by the introduction of analyte. Since there is only one variable, the system is incapable of detecting mixtures. Superior to the RI sensing, the MIR absorption sensing can leverage both changes in the refractive index (*n*) and the extinction coefficient (*k*) such that the two variables can help with detecting a mixture with two unknown analytes. Furthermore, the MIR spectroscopy provides spectral information which can distinguish a mixture with multiple unknows.

**Fig. 8:**
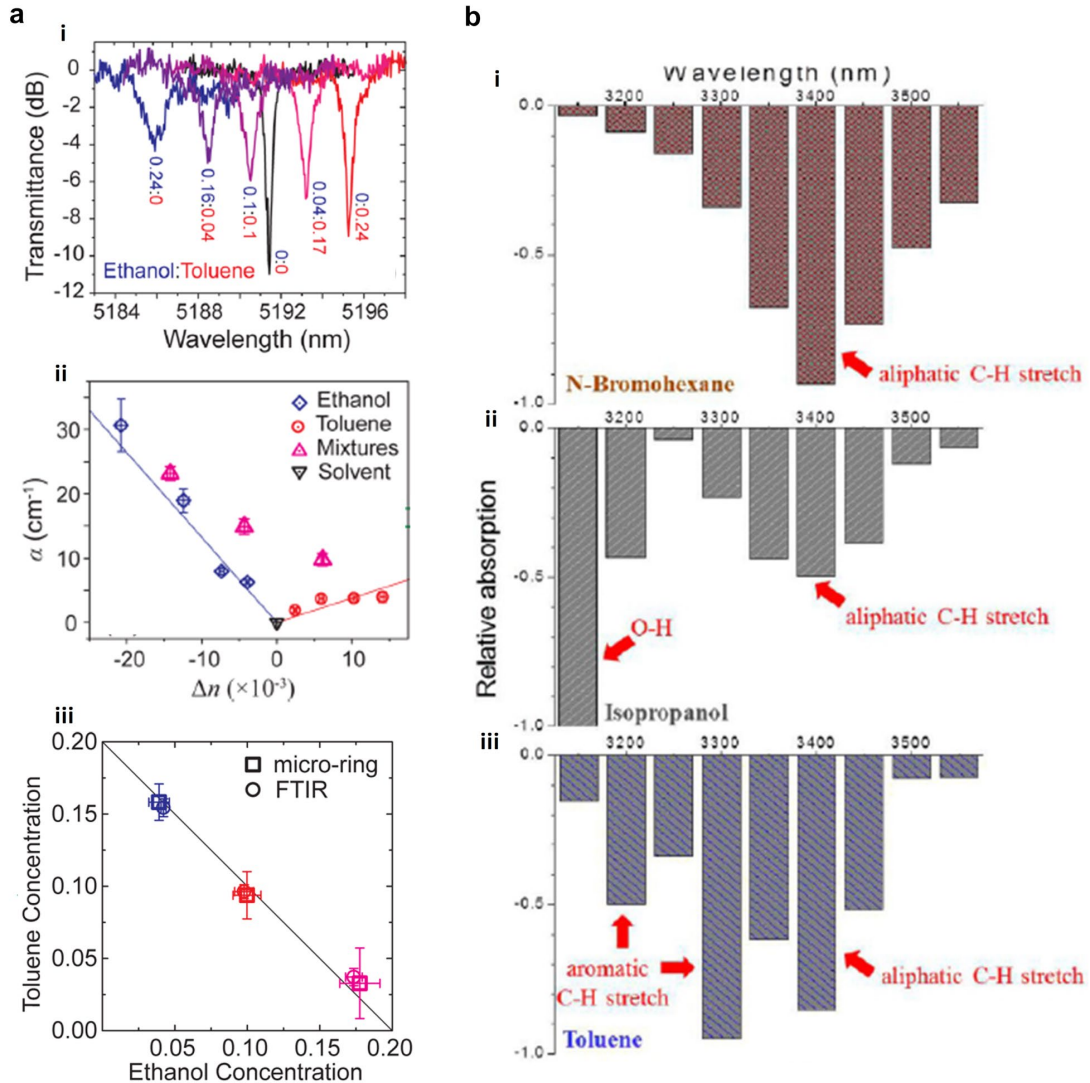
MIR sensing analysis approaches. **a** Mixture sensing analysis using the resonance spectrum of an MRR (Reproduced with permission from [165]), (i) MRR resonance spectrum, (ii) Calculated absorption coefficient from extinction ratio change and refractive index change from resonant wavelength shift, (iii) Individual analyte concentration after linear transformation. **b** Relative absorption spectrum of three chemicals (Reproduced with permission from [156]), (i) *N*-Bromohexane, (ii) Isopropanol, (iii) Toluene

In 2014, a heterogeneously integrated Si nanophotonic MIR spectroscopic sensing device featuring the Si MRR transferred onto a MIR-transparent CaF_2_ substrate was demonstrated [165]. The device works at around 5.2 µm for sensing an ethanol/toluene (C_2_H_5_OH/C_7_H_8_) mixture in cyclohexane (C_6_H_12_). As shown in Fig. 8a (i), the resonance of the ring resonator changes both its wavelength and extinction ratio when mixtures with different C_2_H_5_OH/C_7_H_8_ volume ratios are added. The change of *n* can be extracted from the resonant wavelength shift while the change of *k* can be derived from the extinction ratio change. The measured absorption coefficient *α* (which is solely determined by *k* via *α *= 4π*k*/*λ*), and the refractive index change of the mixtures Δ*n* are presented in Fig. 8a (ii) together with the results from calibration samples containing only C_2_H_5_OH or C_7_H_8_ in C_6_H_12_. The calibration results are fitted well by linear functions while the results from the mixtures fall between the two linear fittings. Therefore, a linear transformation relating α to Δ*n* and the C_2_H_5_OH concentration (*c*_e_) and C_7_H_8_ concentration (*c*_t_) is suggested:

13 $$\left[ {\begin{array}{*{20}c} {c_{e} } \\ {c_{t} } \\ \end{array} } \right] = \left[ {\begin{array}{*{20}c} A & B \\ C & D \\ \end{array} } \right]\left[ {\begin{array}{*{20}c} {\Delta n} \\ \alpha \\ \end{array} } \right]$$

After the empirical coefficient A, B, C, D are determined by the calibration samples, *c*_e_ and *c*_t_ of any C_2_H_5_OH/C_7_H_8_ can be obtained once *α* and Δ*n* are measured. An exemplary result is shown in Fig. 8a (iii), this technique presents a similar accuracy level compared to the commercial Fourier-transform infrared spectroscopy (FTIR) measurement. In 2018, an SWG-based chemical sensor capable of detecting changes in both *n* and *k* was proposed. In this design, an SWG structure is incorporated into the conventional DC [122]. A sharp trough is created in the transmission spectrum due to Bragg diffraction at the Bragg wavelength. By measuring the shift of the Bragg wavelength, which is sensitive to RI change, Δ*n* can be derived. Meanwhile, *k* can be obtained directly by measuring the absorption at the fingerprints of analytes.

One major challenge in the MIR absorption sensing is that many substances have their fingerprints at a common wavelength. This issue has been addressed in 2014 [156]. As presented in Fig. 8b, *N*-bromohexane (C_6_H_13_Br), IPA, and C_7_H_8_ all have strong absorption at 3.4 µm. However, across 3.15 µm to 3.55 µm, C_6_H_13_Br only has one absorption peak; IPA has another absorption peak due to the O–H stretch at 3.15 µm, and C_7_H_8_ has two extra absorption peaks at 3.2 µm and 3.3 µm due to the aromatic C–H stretch. Consequently, by counting the number of strong absorption peaks across 3.15 µm to 3.55 µm, C_6_H_13_Br, IPA, and C_7_H_8_ can be distinguished. Furthermore, based on a similar methodology, by analyzing the ratio of absorption at 3.45 µm to 3.25 µm, the individual concentration of C_2_H_5_OH and acetonitrile (C_2_H_3_N) in C_2_H_5_OH/C_2_H_3_N compound can be determined.

The lists of representative infrared guided-wave nanophotonic biochemical sensors based on RI change and optical absorption are provided in Tables 1 and 2, representatively.

**Table 1:** Summary of representative RI-based infrared guided-wave nanophotonic biochemical sensors

Sensor configuration	Improvement strategy	Material platform	Sensing pathlength (mm)	Bulk sensitivity (RIU^−1^)	Bulk LoD (RIU)	Surface sensitivity	Surface LoD	Refs.
MZI	TM mode	SOI	2	~ 460 × 2π rad	N/A	N/A	~ 6 pg/mm^2^	[29]
MZI	Slot, Vernier effect	SNOI	7	1730 × 2π rad	1.29 × 10^−5^	60 nm/(ng/mm^2^)	0.155 pg/mm^2^	[80]
MZI embedded with PhC nanobeam	Slow light effect	SOI	0.016	1344 rad	N/A	N/A	N/A	[107]
Cascaded MZI and MRR	Vernier effect	SOI	5.4	21,500 nm	N/A	0.5 nm/(ng/ml)	0.1 ng/ml	[119]
Microracetrack resonator	N/A	SOI	Radius 0.005	70 nm	1 × 10^−5^ (i)^b^	10 ng/ml	N/A	[33]
MRR	Photodetector integration	SOI	Radius 0.1	~ 18.8 nm	3.5 × 10^−5^	N/A	N/A	[38]
MRR array	TM mode, temperature compensation	SOI	N/A	135 nm	7 × 10^−7^	N/A	0.3 pg/mm^2^	[68]
MRR	90 nm thick	SOI	N/A	133 nm	~5 × 10^−4^ (i)	N/A	N/A	[71]
Microracetrack resonator	TM mode, 150 nm thick	SOI	5.053	270 nm	1.2 × 10^−3^ (i)	N/A	N/A	[72]
MRR	Suspended	SOI	Radius 0.005	310 nm	N/A	N/A	N/A	[74]
MRR array	Slot, 1310 nm wavelength^a^	SNOI	Radius 0.07	246 nm	5 × 10^−6^	1.3 nm/(ng/mm^2^)	0.9 pg/mm^2^	[77]
MRR	SWG	SOI	Radius 0.01	440.5 nm	~ 3.9 × 10^−4^ (i)	~ 1 nm/nm	N/A	[90]
MRR	Multibox	SOI	Radius 0.03	579.5 nm	1.02 × 10^−3^ (i)	1900 pm/nm	0.313 nm (i)	[92]
Two cascaded MRR	Vernier effect	SOI	2.514	2169 nm	8.3 × 10^−6^	N/A	N/A	[114]
Two cascaded MRR	Vernier effect, suspended	SOI	Radius 0.05	4.6 × 10^5^ nm	4.8 × 10^−6^ (i)	N/A	N/A	[115]
Two cascaded double-MRR	Vernier effect, TM mode	SOI	~ 0.8	24,300 nm	N/A	N/A	N/A	[117]
Three cascaded MRR	Vernier effect	SOI	Radii 0.132 and 0.138	5866 nm	1.3 × 10^−5^ (i)	N/A	N/A	[118]
Microdisk resonator	TM mode	SOI	Radius 0.01	142 nm	6.8 × 10^−4^	TM 3 times of TE	N/A	[70]
Microdisk resonator	TM mode, suspended	SOI	Radius 0.0008	130 nm	8 × 10^−4^ (i)	N/A	N/A	[73]
Microdisk resonator	Horizontal slot	SiN_x_/SiO_2_/SiN_x_ on Si	Radius 0.0075	N/A	N/A	2.5 nm/(µg/ml)	30 ng/ml	[87]
PhC nanobeam cavity array	N/A	SOI	N/A	N/A	N/A	0.35 nm/nm	63 ag (i)	[45]
PhC H0 cavity	N/A	SOI	N/A	176 nm	N/A	N/A	20 pM	[50]
PhC WMC cavity coupled W1 waveguide	Suspended	SOI	N/A	103 nm	2.4 × 10^−3^ (i)	24.7 ± 4.5 nm/pg	490 ± 70 pg/mm^2^ (i)	[52]
PhC L9 cavity	Slot, suspended	SOI	N/A	421 nm	1 × 10^−5^	N/A	N/A	[85]
PhC L55 cavity coupled W1 waveguide	Slow light effect	SOI	0.023	N/A	N/A	N/A	50 fM (3.35 pg/ml)	[102]
PhC L13 cavity coupled W1 waveguide	Slow light effect, defect holes	SOI	N/A	112 nm	1 × 10^−7^ (i)	N/A	1 fM (67 fg/ml)	[105]
PhC hetero cavity	Slot, suspended	SOI	N/A	1538 nm	7.8 × 10^−6^ (i)	N/A	N/A	[83]
PhC W1 waveguide	Slow light effect	SOI	0.02	174.8 nm	1.55 × 10^−4^	0.706 nm/(ng/mm^2^)	2.1 pg/mm^2^	[106]
Bragg grating	Slow light effect	SOI	0.038	150 nm	1 × 10^−5^	N/A	N/A	[58]
Phase-shifted Bragg grating	TM mode, 1310 nm wavelength	SOI	N/A	106 nm	1.62 × 10^−4^ (i)	N/A	N/A	[40]
Phase-shifted Bragg grating	Slot	SOI	0.132	340 nm	3 × 10^−4^ (i)	N/A	N/A	[86]

^a^1550 nm where not specified

^b^denote iLoD. sLoD where not specified

**Table 2:** Summary of representative absorption-based infrared guided-wave nanophotonic biochemical sensors

Sensor configuration	Improvement strategy	Material platform	Analyte	Working wavelength (nm)	Sensing pathlength (mm)	Sensitivity	LoD	Refs.
Straight ridge waveguide	TM mode	GeSbS on insulator	*N*-methylaniline	Around 1500	1000	43.3 dB/vol. %	0.07 cm^−1^ absorption coefficient	[21]
Spiral rib waveguide	N/A	SOI	Glucose	Around 1590	10	N/A	1 mM	[61]
Zigzag ridge waveguide	TM mode	SOI	CH_4_	Around 1651	100	N/A	772 ppmv Hz^−1∕2^	[62]
Spiral ridge waveguide	TM mode, full integration	SOI	CH_4_	Around 1651	300	N/A	92.8 ppmv Hz^−1∕2^	[63]
PhC W0.8 waveguide	Slow light effect, slot	SOI	Xylene	Around 1697	0.3	Refer to Fig. 5 in the paper	100 ppbv	[110]
PhC W1.3 waveguide	Slow light effect, slot	SOI	CH_4_	Around 1665.5	0.3	N/A	100 ppmv	[111]
Two PhC W1 waveguide	Slow light effect	SOI	Xylene, trichloroethylene (TCE)	Around 1644, around 1674	0.3	N/A	1 ppbv xylene, 10 ppbv TCE	[112]
Microtoroid resonator	Cavity enhancement	SiO_2_ on Si	D_2_O	1300	N/A	N/A	1 ppmv	[19]
Microdisk resonator	Cavity enhancement	GeSbS on insulator	*N*-methylaniline	Around 1500	Radius 0.02	N/A	0.02 cm^−1^ absorption coefficient	[22]
MRR	Cavity enhancement, TM mode	SOI	*N*-methylaniline	Around 1500	Radius 0.1 (effective free space path length 5 mm)	N/A	2 nl	[41]
Pedestal waveguide	Pedestal waveguide	SNOI	Glucose	2730–3100/3300–3600	8	N/A	150 mg dl^−3^	[137]
Straight ridge waveguide	N/A	SOI	CO_2_	4260	20	4 × 10^−6^ in transmission	500 ppm	[145]
Straight ridge waveguide	Flexible	AlN on borosilicate	Ethanol, methanol, water	2500–2650	N/A	2% in transmission/1% water	1% water	[149]
Straight ridge waveguide	N/A	Diamond on insulator	Acetone	5780–6350	3	0.13/(mol/l) in absorbance	200 pl	[151]
Straight ridge waveguide	N/A	MCT on CT	Acetone	5780–6350	3	0.023/(mol/l) in absorbance	90 pl/71.1 ng	[152]
Straight SM ridge waveguide	N/A	GOSN	IPA	3650–3900	4	~ 0.0417/vol % in absorbance	5% IPA	[154]
Straight slot waveguide	Slot	Suspended Si	*N*-bromohexane, IPA, toluene	3100–3400	20	N/A	Chemical tracing limit of 10^−3^ volume ratio	[156]
MRR	Cavity enhancement	SOS	N_2_O	4420–4470	Radius 0.12	0.2/ppmv in Q factor	5000 ppmv	[157]
Spiral ridge waveguide	N/A	GeSbS on insulator	CH_4_	3310	20	N/A	2.50%	[160]
Straight ridge waveguide	Paper based fluidics	GOS	Bovine serum albumin	5260–10,000	3	0.005/(mg/ml) in absorbance	0.1 mg/ml	[162]
Straight ridge waveguide	Emitter integration	SOI	CO_2_	4260	N/A	N/A	10%	[163]
Spiral ridge waveguide	Photodetector integration	GeSbS on insulator	CH_4_	3110	1 to 10	0.01/vol % in modal absorption coefficient	1% by volume	[164]
Microdisk resonator	Cavity enhancement	SOCF	Toluene, ethanol, IPA	5140–5260	radius 0.06	N/A	0.05 ng ethanol, 0.06 ng tolune, 0.09 ng IPA	[165]

## Nanoplasmonics enhanced infrared guided-wave nanophotonic biochemical sensors

**Fig. 9:**
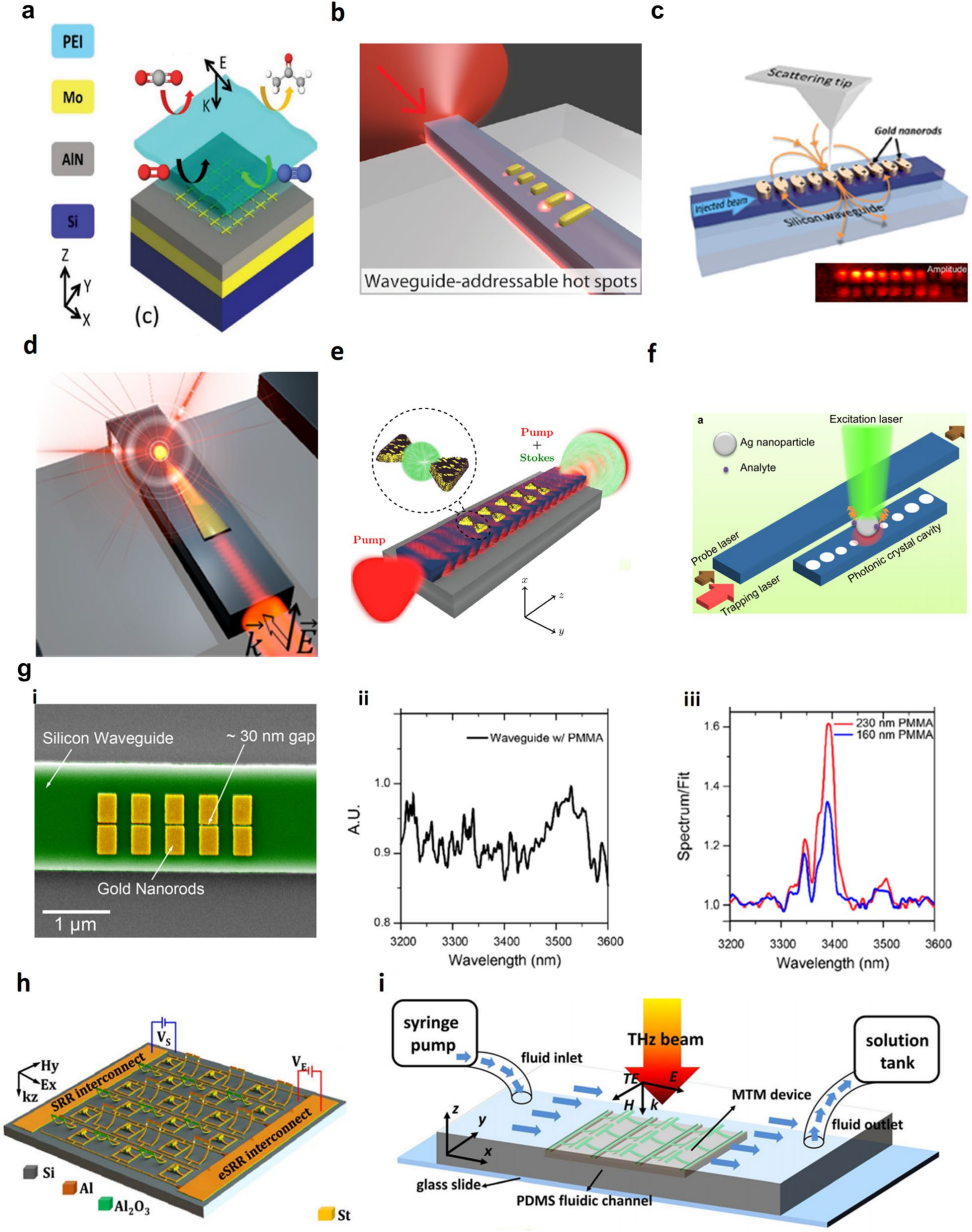
Nanoplasmonics integrated with guided-wave nanophotonics. **a** Nanoplasmonics-based CO_2_ sensor (Reproduced with permission from [174]). **b** Nanoplasmonic hotspots excited by light propagating in a dielectric waveguide (Reproduced with permission from [185]). **c** Near-field nanoplasmonics measurement by the aperture-less scanning optical microscopy (Reproduced with permission from [187]). **d** Nanofocusing on waveguide using a nanoplasmonic tip with 20 nm curvature (Reproduced with permission from [188]). **e** Surface-enhanced Raman Spectroscopy using bow-tie nanoantenna array on waveguide (Reproduced with permission from [189]). **f** Surface-enhanced Raman Spectroscopy with recycling function (Reproduced with permission from [190]. **g** Hybrid nanoplasmonics/photonics system for MIR applications (Reproduced with permission from [191]), (i) device optical image, (ii and iii) measured MIR spectrum in device (ii) without and (iii) with nanoplasmonic structure. **h** MEMS-reconfigurable interpixelated nanoplasmonic metamaterial (Reproduced with permission from [193]). **i** Nanoplasmonic metamaterial-based fluidics for sensing (Reproduced with permission from [209]

The footprints of the current guided-wave nanophotonic biochemical sensors are restricted by the optical diffraction limit [166, 167]. Even in the SOI platform where the refractive index difference is among the largest, the waveguide cross-section still requires a dimension of 1.2 × 0.4 µm^2^ for the operation at 3.8 µm [121]. The waveguide length ranges from several millimeters to tens of centimeters to provide significant light–matter interactions. Nanoplasmonics is a promising candidate to integrate with guided-wave nanophotonic biochemical sensors for miniaturization. Nanoplasmonics is the study of electromagnetic phenomena caused by the collective oscillation of electrons at the surface of nanoscale metallic structures when they are subjected to electromagnetic wave excitation [168–171]. The quantum of the free electron oscillation is called surface plasmon, which has a length scale from 2 to 20 nm limited by the skin depth of metals [172]. Recently, a nanoplasmonics-based CO_2_ sensor has been demonstrated. In a device footprint of only 20 µm by 20 µm, the CO_2_ detection with a detection limit down to 40 ppm was achieved by leveraging the high electric field enhancement by crosswire nanoplasmonic structures together with a thin-film membrane for selective gas sensing and local gas enrichment (Fig. 9a) [173, 174]. Similar nanoplasmonic-enhanced MIR absorption sensing applications have also been demonstrated using bow-tie [175–178] and crooked [179] nanoplasmonic structures. In order to increase the sensing area, a high aspect ratio plasmonic nanotrench structure was proposed. When the sensing area increases by 14.5 times, an over 9% increase in absorption was reported [180]. On top of sensing, low dimensional materials such as graphene have been introduced into the nanoplamonic systems for enhancing plasmonic absorbers [181], enhancing nonlinear optical effects [182], tuning plasmon–phonon coupling [183], and enabling plasmonic modulation [184]. Similar technologies can be utilized in guided-wave nanophotonic biochemical sensors by integrating nanoplasmonics on the sensing waveguide and concentrating a high electric field locally in nanoscale to enhance the sensing performance as well as other applications that require strong light–matter interactions.

As shown in Fig. 9b, the hot spots originated from the nanoplasmonic nanorod structure can be excited by the propagating light in the dielectric waveguide [185, 186]. The near-field of the nanoplasmonic structure can be investigated by an aperture-less scanning near-field optical microscopy to provide the amplitude and phase information of the plasmonic mode (Fig. 9c) [187]. In 2015, an ultra-small hybrid photonics-plasmonic light concentrator for nanofocusing in an integrated Si nanophotonics platform was demonstrated (Fig. 9d) [188]. The measured effective area of the focus spot at the nanotaper tip was only 0.013 µm^2^. And the field concentration factor reaches 4.9 in a sample device with a radius of curvature of around 20 nm at the nanotaper tip. With the successful integration of nanoplasmonics on waveguide together with its high performance in terms of nanofocusing and electric field concentration, the hybrid nanoplasmonics/photonics system is used for surface-enhanced Raman spectroscopy (SERS). As shown in Fig. 9e, bow-tie nanoantenna arrays coated with 4-nitrothiophenol (NTP) monolayer were fabricated on a SiN waveguide [189]. Without nanoantenna, Stokes shift is not visible in the spectrum. As the number of nanoantennas increases, the Stokes shift signal becomes stronger, demonstrating the effectiveness of integrating nanoplasmonics to waveguides for SERS. Compared with the free space Raman scattering of NTP molecule, an 8 × 10^6^ enhancement in the Stokes power was achieved. One drawback of this SERS is the requirement of molecule binding where device surface cleaning and recycling are prohibited. To solve this issue, a dynamic SERS platform consisting of a feeding waveguide to a PhC cavity was proposed [190]. The working mechanism of this platform is demonstrated in Fig. 9f. Due to the strong resonance in the nanocavity, the gradient electric field produces enough optical force to trap the silver nanoparticle bonded with the analyte for SERS. When the light is turned off in the feeding waveguide, the resonance stops so that the trapped silver nanoparticle is flushed away. Turning the laser on helps in trapping a fresh silver nanoparticle for device recycling. Recently there were also progresses in the hybrid nanoplasmonics/photonics system for MIR applications. It was reported in 2018 that the detection of a thin poly(methyl methacrylate) (PMMA) film and an ODT monolayer in an integrated nanoplasmonics-photonics platform with a footprint of only 2 µm^2^ (Fig. 9g (i)) [191]. Comparing Fig. 9g (ii) to Fig. 9g (iii), it is evident that the thin PMMA detection in a small footprint on waveguide is only made feasible with the aid of nanoplasmonics. The tunability of nanoplasmonics can be achieved by incorporating microelectromechanical systems (MEMS) technology [192]. In the terahertz regime, reconfiguration of individual pixels in the nanoplasmonic metamaterial has been demonstrated (Fig. 9h) [193–208]. Furthermore, MEMS-based nanoplasmonics devices have been integrated with microfluidics for sensing applications (Fig. 9i) [209]. It is envisaged that the MEMS tunability and the MEMS-microfluidics integration can be introduced to nanoplasmonics enhanced infrared guided-wave nanophotonic biochemical sensors to enable more versatile applications.

## Infrared guided-wave nanophotonic physical sensors

In addition to the biochemical sensors described in the above sections, infrared guided-wave nanophotonic devices have also been widely utilized for various physical sensing applications, most of which are based on the mature NIR Si nanophotonics platform. Representative reported infrared guided-wave nanophotonic physical sensors are reviewed in this section.

### Temperature sensor

Nanophotonic temperature sensors are based on the combination of TO effect and thermal expansion effect. Hence, both the RI and the structure of nanophotonic device are altered by the temperature variation, leading to the changes in optical signals.

Kim et al. [210] demonstrated a temperature sensor based on the MRR with a sensitivity of about 83 pm/°C through monitoring the resonance shift. Xu et al. [211] improved the sLoD of MRR-based temperature sensor by replacing the measurement approach. Instead of measuring the resonance shift by wavelength scanning, the wavelength was fixed at the point of steepest descent on the sideband of the resonance. As a result, the temperature-dependent resonance shift resulted in a large change in the transmitted intensity. Utilizing this method, a temperature difference down to 80 μK was detected, showing a 13-fold improvement in the sLoD as compared to the wavelength scanning methodology. The Vernier effect by two cascaded MRRs was also employed by Kim et al. [212] to enhance the temperature sensing performance. The two MRRs were designed to have different temperature sensitivities and FSRs by tailoring the in-plane geometric parameters. As a result, both the sensitivity and the sensing range were enhanced, showing an enhancement factor of 6.3 and 5.3, respectively. Klimov et al. [213] demonstrated a Bragg grating temperature sensor by monitoring the band edge shift and showed a sensitivity of about 82 pm/°C. Tao et al. [214] proposed a temperature sensor using a photonic waveguide-based Michelson interferometer (MI). The MI is composed of a DC and two waveguide arms with a mirror at each end. The light is split by the DC and guided into the two arms. After the reflection by the two mirrors, the reflected light beams are recombined through the DC. The temperature change induces the change of the phase difference between the two reflected beams, which can be read out by recording the MI destructive interference wavelength shift. A sensitivity of 113.7 pm/°C was achieved. To better leverage the TO effect, Zhang et al. [215] developed a temperature sensor based on cascaded PhC nanobeam cavities. Two PhC nanobeam cavities with different designs are embedded into the two arms of an MZI. One is a stack width modulated PhC nanobeam cavity covered with SU-8 cladding. As most of the light is confined in the SU-8 cladding, this cavity shows a negative TO coefficient. The other is a parabolic-beam PhC nanobeam cavity, which has a positive TO coefficient since most of the optical mode is confined in the Si core. By measuring the difference in the resonant wavelength shifts of the two cavities, a high sensitivity of 162.9 pm/°C was obtained.

**Fig. 10:**
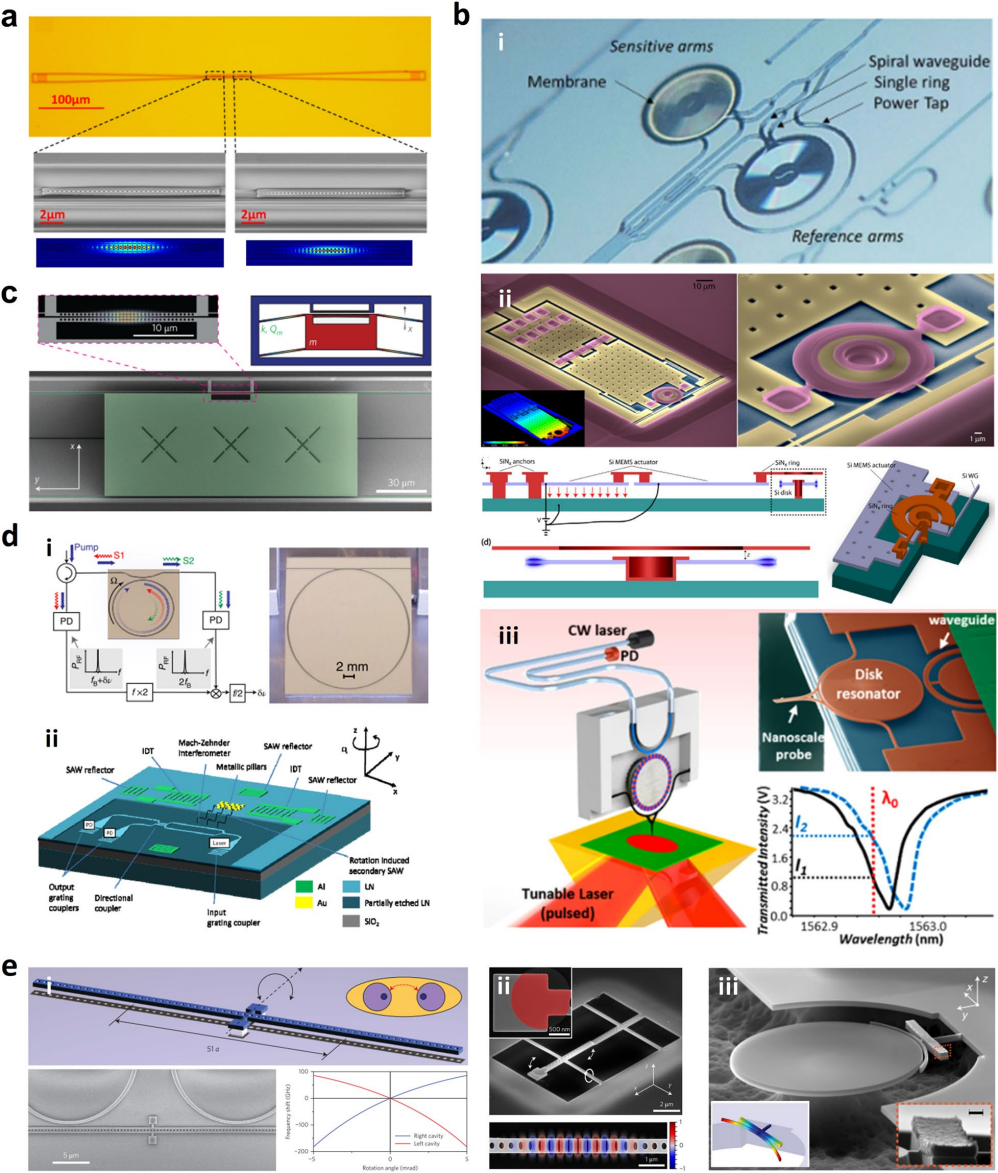
Infrared guided-wave nanophotonic physical sensors. **a** Temperature sensor. Example of simultaneous measurement of solution concentration and temperature using cascaded side-coupled PhC nanobeam cavities (Reproduced with permission from [217]). **b** Force/Pressure/Displacement sensor: (i) MZI pressure sensor (Reproduced with permission from [228]); (ii) force and displacement sensor using coupled Si microdisk resonator and SiN_x_ MRR (Reproduced with permission from [232]); (iii) functional AFM detection using optomechanical transducer (Reproduced with permission from [235]). **c** Accelerometer. Example of optomechanical accelerometer using zipper PhC nanocavity (Reproduced with permission from [236]). **d** Gyroscope: (i) microresonator Brillouin gyroscope based on Sagnac effect (Reproduced with permission from [241]); (ii) acousto-optic gyroscope (Reproduced with permission from [243]). **e** Torque/Magnetic field sensor: (i) torque sensor using nanobeam with two 1D PhC nanocavities (Reproduced with permission from [244]); (ii) magnetic field sensor using split-beam nanocavity (Reproduced with permission from [246]); (iii) magnetic field sensor using coupled optical microdisk resonator and arced torsional mechanical resonator (Reproduced with permission from [248])

As material RIs are dependent on temperature, it is meaningful to integrate the temperature sensor with the RI-based biochemical sensor, so as to decouple the influences of temperature and biochemical analyte on the optical signal change. Kwon et al. [216] demonstrated an MRR-based sensor measuring both the concentration and temperature of a solution. It consists of two MRRs with the same radius consecutively coupled to a bus waveguide, one of which is covered by a cladding and most part of the other uncovered. As a result, the resonant wavelengths of the two MRRs change similarly with the temperature but differently with the concentration. A similar idea was adopted by Xu et al. [68] Real-time measurements showed that the reference resonator covered by a cladding tracked the temperature change without any noticeable time delay, enabling the effective cancellation of temperature-induced shift for biochemical sensing. Liu et al. [217] realized the simultaneous measurement of RI and temperature using two cascaded PhC nanobeam cavities coupled to the same bus waveguide, as shown in Fig. 10a. One of the cavities is an air-mode cavity with more electric field penetrating into the cladding, thus has a higher sensitivity to the variations in solution concentration. The other is a dielectric-mode cavity with the majority of the electric field confined inside the Si core and it is more sensitive to the temperature change. Therefore, the solution concentration (solution RI) and the temperature could be simultaneously extracted in a single measurement.

### Optomechanical sensors

Another major category of nanophotonic physical sensors is the optomechanical sensor, which relies on the interaction between optical field and mechanical objects. The exploration of optomechanical interaction leads to the sensing of various mechanical stimuli, including force, pressure, acceleration, angular velocity, torque, etc. Optomechanical sensors are commonly constructed by freestanding nanophotonic cavities. Various mechanisms can contribute to optomechanical sensing, including optical path length changes and evanescent filed coupling changes induced by the mechanical movement of nanophotonic components, and the microscopic photon-phonon coupling induced by the photoelastic (PE) effect and the moving interfaces (MI) effect. The PE effect is a result of mechanical strains inside the waveguide core which changes the dielectric permittivity of the core material, while the MI effect comes from the dielectric permittivity variation in the vicinity of the core-cladding boundaries caused by their mechanical motion [218, 219].

#### Force/pressure/displacement sensor

Lee et al. [220] proposed a Si nanophotonics based cantilever sensor, where a PhC nanocavity is embedded at the junction edge of a cantilever and the substrate. When the cantilever is deformed, the maximum strain and stress occur at the junction edge, leading to significant changes in the defect length and the modal effective index of the PhC nanocavity. As a result, the resonant wavelength shifts. This sensor could be employed to measure a variety of physical parameters such as force, stress, strain, and displacement. The minimum detectable strain, vertical deflection at the cantilever end, and force load were observed as 0.0133%, 0.37 μm, and 0.0625 μN, respectively [221]. This kind of cantilever sensor could also be surface-functionalized and integrated with a microfluidic channel for biochemical sensing applications, where the selective molecule binding induces the cantilever deflection [222]. Mai et al. [223] compared the sensing performances among the nanocavity and hexagonal nanoring resonators of two different layout configurations. The minimum detectable force and detectable strain were obtained in one of the hexagonal nanoring resonator configurations and were derived as small as 0.0757 μN and 0.0023%. Similar ideas were developed by Li et al. using PhC-based dual-nanoring channel drop filters on cantilevers. Through the optimization of the nanophotonic structures, the minimum detectable force was improved from 37 nN [224] to 7.58 nN [225]. A nanoscale force and pressure sensor based on a hexagonal PhC lattice with a triple-nanoring resonator created at the center of a Si circular diaphragm was also developed, showing a minimum detectable force of 0.847 µN and a minimum detectable pressure of 4.17 MPa [226].

Zhao et al. [227] reported a pressure sensor using an MRR located on a diaphragm. With a pressure applied onto the diaphragm, the diaphragm deforms and changes the radius of the MRR, leading to the resonant wavelength shift. A sensitivity of 1.47 pm/kPa and a resolution of 1.36 kPa were demonstrated. Rochus et al. developed another MZI-based pressure sensor. As shown in Fig. 10b (i), the sensing arm embedded with a spiral waveguide is seated on a membrane. The deformation of the membrane leads to an elongation of the optical path, hence inducing a phase shift in the sensing arm, which is read at the MZI output and converted to the electrical signal by optoelectronic components and readout electronics. A sensitivity of 8 mV/Pa and a resolution of 1.5 Pa were measured [228–230]. The MZI-based pressure sensor was further optimized and demonstrated its use as a microphone [231].

Aksyuk’s group from the National Institute of Standards and Technology developed an optomechanical sensing system, as shown in Fig. 10b (ii). The sensing system consists of a fixed Si microdisk resonator and a suspended SiN_x_ MRR. When the MRR moves close to and interferes with the microdisk resonator in the evanescent field, the modal effective index and thus the resonant frequency of the microdisk resonator are modified, which are measured by a side-coupled waveguide. A displacement sLoD of 4.6 fm/Hz^1/2^ and a force sLoD of 53 aN/Hz^1/2^ were achieved, with only 250 nW optical power launched into the sensor [232]. They developed another optomechanical transducer in which a Si cantilever with a sharp probe tip is separated from a high Q factor microdisk resonator by a nanoscale gap. The cantilever geometry was tailored for both strong optomechanical interactions and applicability to atomic force microscopy (AFM). A displacement sLoD of ~ 4.4 × 10^−16^ m/Hz^1/2^ and bandwidth > 1 GHz, and a dynamic range > 10^6^ was estimated for a 1 s measurement [233]. The cantilever stiffness range compatible with the cavity optomechanical readout scheme was also investigated. A displacement sLoD on the order of 1 fm/Hz^1/2^ could be achieved while the cantilever stiffness was varied over four orders of magnitude (~ 0.01 N/m to ~ 290 N/m), which covers the stiffness range of conventional AFM [234]. Afterward, the functional AFM detection employing such an optomechanical transducer was successfully realized. As illustrated in Fig. 10b (iii), the optomechanical transducer was integrated into a photothermal induced resonance (PTIR) setup. The sample is heated up by a MIR pulsed laser and expands, inducing vibration in the probe with its amplitude directly proportional to the absorption coefficient of the sample. The wavelength of the NIR continuous wave (CW) laser is fixed at the steepest slope point on the resonance side of the microdisk resonator, such that the probe motion modulates the transmitted light intensity. The optomechanical transducer improved the PTIR SNR by ∼ 50 compared with the conventional AFM cantilever [235].

#### Accelerometer

The monitoring of acceleration is indispensable for various applications ranging from inertial navigation to consumer electronics. Although optical detection provides superior resolution, robustness to electromagnetic interference and long-range readout, conventional optical accelerometers are hindered by the low integration level and low bandwidth due to a bulky proof mass. Krause et al. [236] demonstrated an optomechanical accelerometer using a zipper PhC nanocavity integrated with a nanotethered nanogram proof mass with a high mechanical Q factor, as shown in Fig. 10c. The zipper cavity consists of two PhC nanobeams, one attached to the proof mass and the other anchored to the bulk. With the optical cavity field being largely confined to the slot between the nanobeams, the optical resonance frequency is sensitive to the in-plane relative motion of the nanobeams, which is induced by the proof mass displacement caused by in-plane acceleration. A fiber taper waveguide was used to couple light evanescently into and out of the nanocavity. An acceleration resolution of 10 µg/Hz^1/2^ with submilliwatt optical power, a bandwidth greater than 20 kHz and a dynamic range of greater than 40 dB were achieved. Dong et al. [237] reported an optomechanical accelerometer employing MRRs. A nanotethered proof mass is placed between two MRRs and evanescently coupled with them. Differential measurement was taken to double the sensitivity, which reached 3.279 pm/g.

#### Gyroscope

In addition to accelerometers, gyroscopes that measure angular velocities are also essential in commercial and military systems. Optical gyroscopes mostly rely on the Sagnac effect. When the gyroscope rotates, the effective path lengths of the two counterpropagating modes become different, and this difference induces a change of the resonant frequency as well as the phase of each propagating mode. Therefore, the angular velocity can be extracted by measuring the resonant frequency difference or the phase difference between the two modes [238]. Numerous optical passive gyroscopes based on fiber spools and waveguide ring resonators have been demonstrated, showing a superior sensitivity and a bias stability compared with MEMS gyroscopes [239]. Li et al. [240] developed a chip-based, micro-optical gyroscope that uses counterpropagating Brillouin lasers to measure rotations as a Sagnac-induced frequency shift. A single pump wave is evanescently coupled to a high-Q microdisk resonator fabricated of silica on a silicon chip to induce the first Brillouin Stokes wave, which propagates in a direction opposite to the pump wave. Upon further pumping, the first Stokes wave increases in power to a point where it acts to pump a second Brillouin Stokes wave, propagating in a direction opposite to the first Stokes wave, i.e., in the same direction as the pump wave. In the same way, the third Stokes wave is excited by the second Stokes wave and propagates in the same direction as the first Stokes wave. The second and third Stokes waves were selected for the sensing purpose. A minimum detectable rotation rate of 22 deg/h was achieved, which surpasses prior passive micro-optical gyroscopes by over 40-fold. The detection limit could be further lowered by improving the linewidth of the Brillouin laser. Gundavarapu et al. [241] realized a Brillouin laser in a Si_3_N_4_ waveguide bus-coupled MRR with a fundamental linewidth of only ~ 0.7 Hz and demonstrated its application as a gyroscope (Fig. 10d (i)). Khial et al. [242] proposed a reciprocal method to improve the detection limit, where two MRRs with a radius of only 500 μm were employed. By using an MZI and two 50/50 DCs, the MRRs can be fed from two different directions, whereby the desired signal has its polarity flipped but undesirable components such as thermally induced fluctuations and fabrication induced mismatch are common and thus can be attenuated. As a result, the SNR is improved. A smallest recorded phase shift of 3 nrad was achieved, which is 30 times smaller than state-of-the-art miniature fiber-optic gyroscopes.

Mahmoud et al. [243] investigated a novel optomechanical gyroscope by replacing the acousto-electrical detection in the surface acoustic wave (SAW) gyroscope with an acousto-optical detection method, which provides advantages of low noise level, high sensitivity, and stable readout. Figure 10d (ii) schematically illustrates the operation principle. A SAW standing wave pattern is established along the *x*-direction by the two interdigitated transducers (IDTs). The pillars are placed at the anti-nodes of the SAW standing wave pattern and vibrate as driven by the SAW. When an out-of-plane rotation is applied, the Coriolis force is induced on the vibrating pillars in the *y*-direction. The pillars are arranged in a checkerboard configuration such that their constructive interference establishes a secondary SAW in the *y*-direction, which can be directly related to the Coriolis force. The secondary SAW propagates through and is optically read by the MZI based on the acousto-optic effects including PE effect, MI effect, and electro-optic (EO) effect as the SAW generates a moving mechanical strain field as well as a moving electric field due to the piezoelectric effect. The two arms of the MZI are separated by 1.5 times of the SAW wavelength. As a result, when one arm is under compression, the other is under tension, enabling a differential operation to enhance the sensitivity.

#### Torque/magnetic field sensor

Li et al. [244] demonstrate a novel torsional multi-cavity optomechanical system, which consists of a nanobeam inscribed with two 1D PhC nanocavities, one on each side, as shown in Fig. 10e (i). The optical force generated by photons inside the nanocavities excites the rotational motion of the nanobeam, which asymmetrically modulates the frequencies of both nanocavities. The optomechanical system realized a torque detection limit of 9.7 × 10^−21^ Nm/Hz^1/2^. Wu et al. [245] developed an optomechanical torque sensor based on a split-beam nanocavity. The split-beam nanocavity supports high optical Q factor modes localized in the gap between the two suspended PhC nanobeams, which serve as optical mirrors. One of the mirrors is anchored by single mechanical support and able to rotate, while the other is anchored in three sections and only able to bend at the end close to the gap like a cantilever. Both dissipative and dispersive optomechanical couplings contribute to the sensing application. The mechanical motion of the mirrors modifies the mirror gap, effectively changing the nanocavity length, resulting in a dispersive coupling to the nanocavity optical frequency. The strong dependence of the nanocavity internal photon decay rate on the mirror gap results in a dissipative optomechanical coupling. Additional dissipative coupling arises when the motion of the mirrors modulates the nanocavity external photon decay rate into an external coupling waveguide. Through the optimization of both couplings, torque detection limits of 1.2 × 10^−20^ Nm/Hz^1/2^ in ambient conditions and 1.3 × 10^−21^ Nm/Hz^1/2^ in low vacuum were achieved. Later, they demonstrated torque magnetometry and radiofrequency (RF) magnetic susceptometry in a similar split-beam nanocavity optomechanical torque sensor supporting a permalloy island, as shown in Fig. 10e (ii). A coil below the device was used to create an RF magnetic field in the *z*-direction, so as to rotate the movable mirror around the *y*-axis. The exquisite torque detection sensitivity enables fast acquisition of the unique net magnetization and RF-driven responses of single mesoscopic magnetic structures in ambient conditions, enabling the measurement of magnetic hysteresis and susceptibility [246]. Kim et al. [247] developed another optomechanical torque sensor based on an optical microdisk WGM resonator with a 100 nm gap to a torsional mechanical resonator composed of a torsion rod and a pair of paddles. With an optomechanical noise floor down to 7 fm/Hz^1/2^, torque as little as 4 × 10^−20^ Nm could be detected. They further investigated the magnetic actuation of a similar microcavity optomechanical torque sensor with a microdisk resonator coupled to an arced torsional resonator integrated with a trilayer ferromagnetic needle, as shown in Fig. 10e (iii). The torque along the *y*-axis was induced by a magnetic field along the *z*-axis. As a magnetic field sensor, a linear responsivity of 0.134 ± 0.003 rad/mT and a detection limit of 150 nT were obtained [248]. Du et al. [249] demonstrated a magnetic field sensor based on a pair of suspended coupled PhC nanobeam cavities. One of the cavities is connected with feeding waveguides for light input and output. The other cavity is integrated with an array of suspended Si nano bridges with gold nanowires deposited on top. With a current flowing through the nanowires and a horizontal magnetic field perpendicular to the current, an out-of-plane Lorentz force is generated, which induces an offset variation between the two coupled cavities, resulting in a resonance shift of a selected supermode of the coupled cavities. After converting the optical output signal into an electrical signal via a photodetector, a sensitivity of 22.9 mV/T and a resolution of 48.1 µT/Hz^1/2^ were obtained.

## Conclusions

Recent progress of infrared guided-wave nanophotonics as promising technologies for various biochemical and physical sensing applications are reviewed in this paper. Compared with the detection based on other physical properties, optical detection provides advantages of high sensitivity, low detection limit, low crosstalk, strong multiplexing capability, and resilience to electromagnetic interference. In comparison with conventional optical sensors based on fibers, free-space resonators, etc., the guided-wave nanophotonics technologies offer promising potential for sensor miniaturization and on-chip integration. The research and development milestones of infrared guided-wave nanophotonic sensors surveyed in this review help us to foresee future development directions as the next four points:

First is the extension of working wavelengths to longer wavelengths. As reviewed in Sect. 4, there is a trend to extend the working wavelengths of nanophotonic biochemical sensors from the NIR to the MIR, in order to take full advantages of absorption spectroscopy. However, as indicated in Fig. 5, the wavelength extension much relies on various materials that are not CMOS-compatible, especially in the wavelength range beyond 5 µm, which severely limits the popularization of MIR absorption-based sensors. Si with its transparency window up to 8 μm is still preferred considering its mature fabrication technologies and abundant knowledge bases. The strong absorption losses caused by the substrate materials limit the bandwidths of various Si nanophotonics platforms including SOI, SOS, and silicon-on-nitride (SON), making them unable to make full use of the whole transparency window of Si. To remove this limitation, other Si nanophotonics platforms including freestanding configurations based on suspended membrane and suspended SWG, pedestal waveguides, silicon-on-porous-silicon (SiPSi), and silicon-on-calcium-fluoride (SOCF), have been proposed. However, demonstrations of devices on these platforms in the region of wavelength longer than 5.2 µm are still lacking [132, 133]. Therefore, there is still a large space for the development of MIR Si nanophotonics. In addition to Si, other CMOS-compatible materials with wider transparency windows, such as AlN with transparency window spanning from 0.2 to 13.6 μm, have been attracting more and more research interest as they are promising complements to Si nanophotonics [143, 144].

Second is the improvement of sensing performances. On the one hand for environmental monitoring, the threshold limit value determined by the international environmental standards is at ppb to ppm level [250]. On the other hand for human disease detection through human breath, the detection limit is also at ppb-ppm level [251]. Fundamentally the improvement comes from the enhancement of optical performances of the guided-wave nanophotonic devices, such as reducing the propagation loss and insertion loss, increasing the Q factor, enhancing the electric field, etc., which will also facilitate denser integration, more robust and more practical device demonstrations. In particular, absorptive thin films can be coated on the device surface in order for gas enrichment. As the fabrication technology advances, integrating nanoplasmonics has been proved as an effective approach to improve the sensitivity while shrinking the device footprint. Moreover, another sensing performance in compelling need for improvement is the multiplexing capability. Despite the intrinsic selectivity of absorption spectroscopy, the multiplexed detection may be hindered by the weak absorption, the overlapping absorption bands due to the similar molecular compositions and structures, and the interference among adjacent absorption bands. Although the analysis approaches discussed in Sect. 4.3 have been employed and successfully distinguished the ingredients in tertiary mixtures, they require additional manual analysis and may not work for more complicated mixtures. Artificial intelligence (AI) techniques, as emerging and appealing methodologies for data analysis, have been very recently utilized to deal with the raw data and enhance the multiplexing capability of nanophotonic sensors [252, 253]. In addition, AI techniques have also been employed for the inverse design of nanophotonic devices. After being trained with random initial populations of nanostructure geometries and their known spectra, the deep neural networks (DNNs) are able to predict the spectral response of new nanostructure designs, as well as design new nanostructures based on the wanted spectral response [254, 255]. Moreover, it has been demonstrated that the DNN itself can be realized by nanophotonic circuits [256]. Therefore, it is anticipated that AI techniques will bring nanophotonic sensors to a brand new era.

Third is the improvement on the integration level. The monolithic integration of light sources and photodetectors with sensors is essential for the realization of a portable on-chip sensing system. As reviewed above, in the NIR the waveguide sensor can be conveniently integrated with III–V lasers and photodetectors. However, the integration becomes challenging in the MIR due to the lattice mismatch between Si and traditional MIR detection materials such as HgCdTe, III–V, and II–VI. 2D materials, such as graphene and black phosphorus, are promising alternatives to address this issue. Their integrations with Si waveguides have been demonstrated [131, 257, 258]. Nevertheless, sensing applications based on such MIR integrated devices have not been demonstrated yet.

Last but not least is the unit standardization of sensing performance parameters. As mentioned in Sect. 2 and as clearly seen in Tables 1 and 2, the units of the sensitivities and the LoDs have not been uniformed, even among the sensors based on the same mechanism. However, the unit standardization of the figure of merits is an important step for the commercialization of the nanophotonic sensors, as it enables fair comparison among different sensors and provides a guideline for sensor development [259, 260].

## Data Availability

The datasets used and/or analysed during the current study are available from the corresponding author on reasonable request.

## Abbreviations

IoT: Internet of Things
Si: Silicon
CMOS: Complementary metal–oxide–semiconductor
PIC: Photonic integrated circuit
NIR: Near-infrared
MIR: Mid-infrared
LoD: Limit of detection
RI: Refraction index
MRR: Microring resonator
RIU: Refractive index unit
sLoD: System LoD
iLoD: Intrinsic LoD
Q factor: Quality factor
ChG: Chalcogenide
MZI: Mach–Zehnder interferometer
SOI: Silicon-on-insulator
IgG: Immunoglobulin G
WGM: Whispering gallery mode
FWHM: Full width at half maximum
Ge: Germanium
SNR: Signal-to-noise ratio
FSR: Free spectral range
PhC: Photonic crystal
1D: One-dimensional
2D: Two-dimensional
TDLAS: Tunable diode laser absorption spectroscopy
SC: Supercontinuum
TE: Transverse electric
TM: Transverse magnetic
MPW: Multi-project wafer
DAPK: Death-associated protein kinase
DSHP: Double-slot hybrid plasmonic
SWG: Subwavelength grating
anti-BSA: Anti-bovine serum albumin
TCE: Trichloroethylene
MMI: Multimode interference
DC: Directional coupler
TO: Thermo-optic
SNOI: Silicon nitride-on-insulator
SOS: Silicon-on-sapphire
GOI: Germanium-on-insulator
GOS: Germanium-on-silicon
GOSN: Germanium-on-silicon nitride
(SiGe)-on-Si (SGOS): Silicon–germanium alloy
AlNOI: Aluminum nitride-on-insulator
MCT-on-CT: Mercury cadmium telluride-on-cadmium telluride
LWIR: Long-wave infrared
PEI: Polyethylenimine
PGMA: Poly(glycidyl methacrylate)
PDMS: Polydimethylsiloxane
FTIR: Fourier-transform infrared spectroscopy
SERS: Surface-enhanced Raman spectroscopy
NTP: Nitrothiophenol
PMMA: Poly(methyl methacrylate)
MEMS: Microelectromechanical systems
MI: Michelson interferometer
PE: Photoelastic
MI: Moving interfaces
AFM: Atomic force microscopy
PTIR: Photothermal induced resonance
CW: Continuous wave
SAW: Surface acoustic wave
IDT: Interdigitated transducer
EO: Electro-optic
RF: Radiofrequency
SON: Silicon-on-nitride
SiPSi: Silicon-on-porous-silicon
SOCF: Silicon-on-calcium-fluoride
AI: Artificial intelligence
DNN: Deep neural network
